# The Political Competence Scale for Nurses (PCS-N): Instrument Development and Psychometric Evaluation

**DOI:** 10.1155/jonm/4683994

**Published:** 2025-05-19

**Authors:** Nam Kyung Han, Gwang Suk Kim, Sunah Kim, Tae Wha Lee, Woojin Chung

**Affiliations:** ^1^Department of Nursing, Gyeongbuk College of Health, Daehakro 168, Gimcheon 39525, Gyeongbuk, Republic of Korea; ^2^Mo-Im Kim Nursing Research Institute, College of Nursing, Yonsei University, 50-1 Yonsei-ro, Seodaemun-gu, Seoul 03722, Republic of Korea; ^3^Department of Biohealth Industry, Yonsei University Graduate School of Transdisciplinary Health Sciences, 50-1 Yonsei-ro, Seodaemun-gu, Seoul 03722, Republic of Korea

**Keywords:** competence, measurement, nurses, politics, validation study

## Abstract

**Background:** Few tools are available to measure nurses' political competence, and the existing ones have limitations in reflecting the multidimensional factors of competence. This study developed a multidimensional political competence measurement tool—the Political Competence Scale for Nurses (PCS-N)—and assessed its validity and reliability in measuring nurses' political competence levels.

**Methods:** This methodological study was based on the tool development and tool-test stages suggested by DeVellis.

**Results:** The validity and reliability of the PCS-N were established. The PCS-N was tested for construct validity through content validity, item-total correlations of preliminary items, and exploratory factor analysis. The PCS-N comprises 35 items across four factors: political knowledge, political efficacy, political interaction, and political activity. The suitability of this measurement tool was established through construct validity and confirmatory factor analysis. Concurrent validity was verified and was significantly correlated with existing political science measurement tools, political efficacy, and political interest (*r* = 0.511, *p* < 0.001). Internal consistency reliability (Cronbach's *α* = 0.951) and test–retest reliability were also established, confirming the stability of the PCS-N.

**Conclusions:** The PCS-N can be used to evaluate nurses' political competence and provide a basis for constructing education and training programs to strengthen political competence and evaluate their effectiveness.

## 1. Introduction

Nurses' political competence is low in most countries, resulting in inadequate participation in healthcare policy reforms and political activity [1–6]. The reasons for low political participation among nurses include a lack of interest in politics [6–8], time and resource constraints owing to poor work environments [2, 9], a lack of formal or nonformal education in politics or healthcare policy that could enhance political competence [2, 7, 8, 10], and a lack of support from nursing representative organizations [2]. Therefore, nurses' political competence should be strengthened to encourage their political participation and intervention in healthcare policies that affect public health advocacy and working environments [11–14]. It is necessary to scientifically and objectively identify nurses' competence. A measurement tool whose validity and reliability have been strictly verified makes this possible.

Political competence has been conceptualized by many political scientists since Easton [15] first defined politics in 1953 as the ability to influence the authoritative distribution of social values within governmental organizations. The concept of political competence is somewhat vague and has been used interchangeably with concepts such as political efficacy, political knowledge, political intelligence, political interest, civic competence, and policy competence, as there is no consensus among scholars [16]. Political competence encompasses the attributes of political efficacy, including the perception and attitude that one can influence the policymaking process [11] and promote effective political activities [17]. It also includes the ability to understand and critically accept political information [18, 19], analytical ability [20], and activities through political interactions in the community and legislative settings [12–14, 18, 19, 21].

There have been some studies on the concept development of nurses' political competence [1, 10, 22, 23], but there is no agreed-upon concept among nursing scholars. In prior research, the conceptual dimensions of nurses' political competence presented in nursing include nursing expertise as valued currency, opportunities created through networking, powerful persuasion, commitment to collective strength, strategic perspectives, and perseverance by Warner [10]; deep knowledge, political antenna, and power exercise of nurses' political competence by O'Grady and Johnson [22]; learning to know, learning to be, learning to live together, and learning to do by Melo et al. [23]; and political knowledge, political efficacy, political interaction, and political activity by Han and Kim [1]. Among them, the conceptual dimensions of Warner [10], O'Grady and Johnson [22], and Melo et al. [23] include political knowledge, political interaction, and political activity but not political efficacy; while the dimensions derived by Han and Kim [1] include all the various multidimensional political competence concepts proposed in political science—political knowledge, political efficacy, political interaction, and political activity. Regarding research methodology, Warner [10] explored only empirical indicators without a literature review, and O'Grady and Johnson [22] presented only the results of a literature review centered on nursing without going through an empirical stage. In addition, Melo et al. [23] analyzed a literature review and empirical indicators, but there were limitations in identifying clear dimensions and attributes because the analysis was based on search engines such as Liliacs, SciELO, and Medline and only documents published during a limited period from 2001 to 2013. Han and Kim's [1] concept of nurses' political competence, based on the hybrid model of Schwartz-Barcot and Kim [24], has the strength of integrating systematic reviews of all literature published before 2018 through more than 10 databases, including political science and nursing and empirical analyses of nursing political activists to address the limitations of concept development research in nursing. Therefore, according to Han and Kim [1], nurses with high political competence can influence healthcare policy decision-making through political interactions and activities based on political efficacy and knowledge.

The scientification of politics, which is related to the measurement of political competence, began in earnest in the 1950s and the 1960s by scholars such as Easton [25] and was centered on political analysis under the influence of American behaviorism, a school of psychology that studies only observable and measurable human behavior [26]. A tool mainly used in political science is a similar concept to political competence, but with a single dimension, such as political efficacy [27–29], political interest [30, 31], and political trust [28].

In nursing, there is no standardized tool agreed upon among nursing scholars to measure nurses' political competence, and there is the Political Astuteness Inventory (PAI) checklist by Clark [32] that measures similar concepts. Clark's [32] measurement tool comprises 40 items including voting behavior, participation in professional organizations, awareness of health policy issues, knowledge of legislation and policy processes, knowledge of legislators, and involvement in political processes. Although it can measure political knowledge, interactions, and activity, it has limitations in measuring political efficacy. Thus, as the measurement tools used in political science and nursing have limitations in reflecting all multidimensional concepts, it is necessary to develop a tool that can measure nurses' multidimensional political competence.

Accordingly, this study developed a tool to measure nurses' multidimensional political competence using Han and Kim's concept [1]. This study aimed to verify the validity, reliability, and model fit of the developed tool. This study can be used to objectively measure nurses' political competence and provide an academic foundation for developing education and training programs to strengthen nurses' political competence.

## 2. Materials and Methods

### 2.1. Research Design

**Figure 1:**
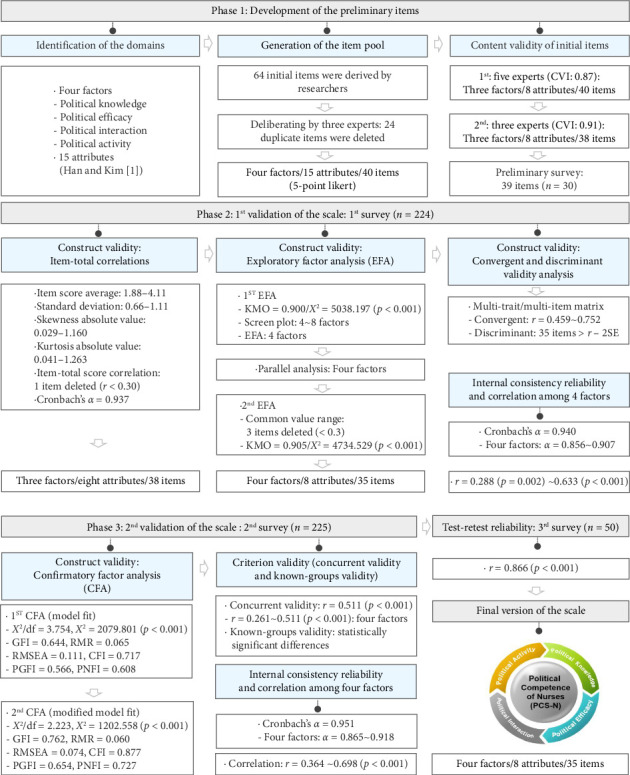
The tool development process based on DeVellis' scale development phase [33]. Abbreviations: CFI, comparative fit index; CVI, content validity index; GFI, goodness-of-fit index; KMO, Kaiser–Mayer–Olkin; PGFI, parsimonious goodness-of-fit index; PNFI, parsimonious normed fit index; RMR, root mean residual; RMSEA, root mean square error of approximation.

This methodological study followed the scale development procedure presented by DeVellis [33], which consisted of the development of an instrument to measure nurses' political competence and psychometric tests of the developed scale's reliability and validity. First, the preliminary item development stage sequentially identified the domains, generated an item pool, tested the content validity of the initial items with experts, and conducted a preliminary survey of the developed items. Second, the researchers performed the construct validity of the scale using the first survey, including item-total correlations, exploratory factor analysis (EFA), and measuring convergent and discriminant validity, followed by verifying the internal consistency reliability of the tool. In the final stage, researchers validated the model fit with construct validity and confirmatory factor analysis (CFA) through a second survey, followed by validating criterion validity using concurrent and known-group validity and testing internal consistency reliability and inter-factor correlations. The final instrument was determined after a third survey to validate the test–retest reliability of the final selection of items (Figure 1).

### 2.2. Scale Development

#### 2.2.1. Conceptual Framework

The concepts and indicators of Han and Kim [1], who presented a multidimensional conceptualization of nurses' political competence, were used as the conceptual framework to develop a measure of nurses' political competence. It comprises four dimensions (political knowledge, political efficacy, political interaction, and political activity), 15 attributes, and corresponding indicators. Political knowledge included political information, political knowledge, and systematic analysis ability; political efficacy included internal and external political efficacy and self-pride of the nursing profession; political interaction included organizations and service activities, networking, and persuasive power; and political activity included political expression, assertive action, political leadership, political advocacy, political participation, and policy intervention [1] (Supporting Table A1, Supporting Figure A1).

#### 2.2.2. Content Validity Test and Preliminary Scale Composition

The initial items were derived from researchers and three experts, including professors with rich backgrounds in political activities, policy interventions, qualitative research, and measurement tool development. The items were written as questions, and a five-point Likert scale (1 = *strongly disagree*, 2 = *disagree*, 3 = *average*, 4 = *agree*, and 5 = *strongly agree*) was used as the response scale. The content validity test of the initial items was conducted over two rounds from July 30 to September 6, 2018, by eight experts, including professors with extensive experience in tool development and political activities, social psychology professors, nurses, and civic society activists, based on Lynn's [34] criterion that 3–10 experts are appropriate. Next, a preliminary survey was conducted to determine whether the items of the completed preliminary tool accurately represented the readability of the vocabulary, grammar, and attributes. A preliminary survey was conducted with 30 nurses who had been working for more than three years in a medical institution or community according to Lynn's [34] criterion that 20–50 were appropriate. A total of 39 items were developed as the final preliminary questionnaire.

### 2.3. Participants for Psychometric Tests

Nonprobability convenience sampling was conducted for the participants to test the preliminary items by considering the distribution of nurses by region and institution who have been working in hospitals, community health institutions, and educational institutions nationwide by referring to the personnel data of the Ministry of Health and Welfare's nursing workforce data in South Korea. Specifically, it reflected the distribution of nursing staff in five provinces across the country since 49.6% of nursing staff work in medical institutions and approximately 25% work in public health institutions under the government. The selection criteria were nurses working in medical institutions with 100 or more beds, health institutions such as public health centers and the National Health Insurance Corporation, and educational institutions such as universities and secondary education institutions in the five provinces nationwide as of the start date of this study; and nurses who understood the purpose and intent of this study, agreed to participate in the study, and had at least three years of experience working in their current workplace. According to the criteria of Tinsley and Tinsley [35], the number of participants for validity, reliability, and CFA of the items test should be five times or more and at least 200 participants; 236 participants were selected for this study considering the 39-items preliminary tool and a nonresponse rate of 10%–20% in most previous studies (Supporting Table A2).

### 2.4. Ethical Considerations and Data Collection

The researchers collected the data after receiving institutional approval (Y-2018-0071) from the Research Review Committee of the Y Medical Center in South Korea for the ethical protection of human research participants. Prior to data collection, the research purpose was fully explained to the institutions and the research was conducted with their cooperation. The recruitment notice was posted on the institution's online bulletin board and participants who voluntarily applied for participation met face to face. The study purpose was thoroughly explained, and informed consent was obtained. In addition, it provided participants with the process and contents of the research, the right to refuse participation in this study, the possibility of voluntarily discontinuing participation, the protection of personal information, and the guarantee of anonymity and that it would not be used for any purpose other than this research. The self-administered questionnaires were completed in a separate quiet room provided by the organizations where the participants worked. Completed questionnaires were sealed in envelopes and collected by the researchers to ensure confidentiality. The collected data were stored on a computer with limited access and lock settings, and a small gift was provided to the participants.

The survey was conducted in three rounds to ensure rigorous validity and reliability; different nurses from new organizations were selected for each round based on the selection criteria to avoid affecting the integrity of the data. The first survey data collection for item-total correlations, EFA, convergent and discriminant validity, and internal consistency reliability tests were conducted from September 13 to October 5, 2018, targeting 224 participants, after excluding missing values and unqualified participants (Supporting Table A3). Subsequently, the second questionnaire data collection for criterion validity, internal consistency reliability, concept validity, and CFA test was conducted from October 29 to November 18, 2018, targeting 225 participants and excluding missing values (Supporting Table A4). Lastly, the third survey data collection to verify the test–retest reliability of the tool was conducted from November 12 to November 27, 2018, 14 days after the survey was completed, targeting 50 participants who agreed to the survey among the second survey participants.

### 2.5. Data Analysis

The data collected in this study were analyzed using SPSS 22.0 and AMOS 22.0 (IBM Corp., Armonk, NY, USA). The content validity of the tool was evaluated using the item-content validity index (I-CVI) with a four-point Likert scale by the expert group. The CVI value was composed mainly of items of 0.80 or higher [36], but if it was less than 0.80, it was modified or deleted through expert discussion to confirm the constituent factors and preliminary items.

Item-total correlations were conducted to test the construct validity of the discriminative power of the preliminary items and the elimination of similar items. Ferketich's criterion [37] was applied to assess corrected item-total correlations and alpha if an item was deleted. According to this criterion, an acceptable range for the corrected item-total correlation is *r* = 0.30–0.70. Items with correlations below *r* = 0.30 may reflect a separate concept within the scale, while those above *r* = 0.70 may represent redundancy or duplication. In addition, EFA was conducted as a method of assessing construct validity by identifying underlying factors that define the concept through statistical analysis. First, the Kaiser–Mayer–Olkin (KMO) and Bartlett's sphericity tests were performed to determine whether the collected data were suitable for EFA. According to the criterion of Kaiser [38], KMO 0.70–0.79 is classified as medium level, 0.8–0.89 as compliance level, and 0.9 or more as high level. EFA uses principal factor analysis and Varimax orthogonal rotation, a factor rotation method, to extract factors by applying the criterion of Peters et al. [39], which suggests that an eigenvalue of one or more, a commonality of 0.40 or more, and a factor loading of 0.50 or more are appropriate. When factor extraction is performed based on Kaiser's criterion, which has an eigenvalue of one or more, there are disadvantages in that subjectivity may be involved when too many factors are derived or when the slope change of the scree plot is nonsignificant [40]. Therefore, a parallel analysis [41, 42], which is the most accurate factor extraction method, was also used. Parallel analysis was analyzed based on the criterion by Costello and Osborne [43]—that it is desirable to have five or more items with a factor loading value of ± 0.50 or more for each factor. A multitrait/multi-item matrix analysis was conducted to test convergent validity, which can identify the relationship among the items developed and the subscales convergent validity was analyzed by controlling overlapping items among multiple items belonging to each factor and then applying the criterion that it is met when the correlation coefficient with the item is at least 0.40 [44]. Discriminant validity applied the criterion that the item is suitable if it is larger than the standard error of twice the correlation coefficient with other subscales to which the item does not belong and if more than 80% of the number of items presented is appropriate [44]. The internal consistency reliability of the tool was assessed using Cronbach's alpha. According to the criterion proposed by Nunnally and Benstein [45], a value of 0.70 or higher is considered acceptable for newly developed tools. Next, a CFA was conducted to evaluate construct validity and model fit. For construct validity, the standardized factor loadings were examined, applying the criterion of Hair et al. [46], which suggests that a standardized loading of 0.50 or higher is appropriate. For model fit in the CFA, various indices were used: *χ*^2^ and *χ*^2^/df, along with the goodness-of-fit index (GFI) as absolute fit indices evaluating overall goodness-of-fit; the root mean square error of approximation (RMSEA) and root mean residual (RMR), which consider model simplicity; the comparative fit index (CFI), which is a relative fit index that evaluates model fit to the base model; and the parsimonious GFI index (PGFI) and parsimonious normed fit index (PNFI) as indices reflecting model simplicity and fit.

After the model fit test, Cronbach's alpha was recalculated to assess the internal consistency reliability of the tool. Criterion validity was analyzed using concurrent and known-group validity tests. The concurrent validity test was performed using instruments from political science to analyze correlations with existing tools, as there were no existing multidimensional instruments for political competence in the nursing field. Therefore, the correlations between the measurement tool developed in this study and the political efficacy and political interest tools used in political science were analyzed using Pearson's correlation coefficient.

The political efficacy scale comprises 11 items [28], developed based on experimental research at the National Election Studies (NES) at the University of Michigan. The political interest tool comprises 14 items [47], also developed based on the NES at the same university. In addition, known-group validity was analyzed using the group comparison method by independent *t*-test and analysis of variance.

To identify the tool's stability, the test–retest reliability was analyzed using Pearson's correlation coefficient for respondents who could be paired with the survey results based on participants' general characteristics. As the test criterion of DeVon et al. [48], a correlation coefficient of 0.70 or higher is acceptable as a stable tool.

## 3. Results

### 3.1. Development of the Preliminary Items: 64 Items ⟶ 40 Items ⟶ 39 Items

#### 3.1.1. Item Pool Generation, Content Validity, and Preliminary Survey

To measure nurses' political competence, the researchers derived 64 initial items based on Han and Kim's conceptual indicators [1] for measuring nurses' political competence. To minimize the reflection of subjectivity in item composition and maintain objectivity during the item derivation process, sentences of indicators suggested by Han and Kim [1] were used as much as possible. Three experts further deliberated on the 64 initial items to determine the accurate reflection of the conceptual indicators and the completeness of the sentences. Consequently, 24 duplicate items that did not accurately reflect the indicators were deleted, and the sentences were revised to 40 items.

Expert content validity tests were conducted three times for the 40 preliminary items. The results of the first content validity test showed that the average value of the CVI of all items was 0.87, two items had an I-CVI index of less than 0.80, and 38 items were derived by deleting or modifying some items. Moreover, among the items with a CVI index of 0.80 or higher, the sentences of some items were modified to reflect political competence (Supporting Table A5). In addition, based on experts' suggestions, similar subitems from the four factors were reorganized into three factors: political knowledge, political interaction, and political activity, and the subattributes of the existing 15 attributes were reorganized into eight attributes. In the second content validity test for the revised 38 items, the average value of the CVI of all items was 0.91, and there were three items with an I-CVI of less than 0.80. Regarding the three items with an I-CVI index of less than 0.80, the researchers discussed them again with experts and decided to retain the items because all three items are related to political efficacy, which is an important political competence indicator in political science. In addition, one item containing two questions was separated into two, and a preliminary tool of 39 items was derived by adding one item (Supporting Table A6).

Next, a preliminary survey was conducted with 30 participants to review the readability and clarity of the 39 preliminary items. The survey items were measured on a four-point scale and the average political competence score of the nurses who participated in the survey was 2.90 ± 0.80 (Supporting Table A7). The Cronbach's *α* value, indicating internal consistency reliability, of the preliminary items was 0.940. In addition, the average time required to complete the questionnaire was 10.63 ± 3.84 min, the appropriateness of the font size was 3.33 ± 0.55, the appropriateness of the item length was 3.17 ± 0.46, and the appropriateness of the response score size (five-point scale) was 3.23 ± 0.50. It was determined that no items were incomprehensible or needed to be deleted; therefore, 39 preliminary items were maintained (Supporting Table A8).

### 3.2. Primary Tool Test (First Survey): Preliminary 39 Items ⟶ 38 Items ⟶ 35 Items

#### 3.2.1. Construct Validity: Item-Total Correlations of Preliminary Items

The average scores for the 39 preliminary items ranged from 1.88 to 4.11. The absolute value of skewness ranged from 0.029 to 1.160, and the kurtosis value ranged from 0.041 to 1.263, which satisfied the criterion of normality of data suggested by Curran et al. [49] when the absolute value of skewness is less than two and the absolute value of kurtosis is less than four. As a result of analyzing the corrected item-total coefficient, the item “I participated in all elections in the last 2 years (*r* = 0.168)” was deleted because it was less than *r* = 0.30. Moreover, the items with *r* = 0.700 exceeded two items: “I have a vision for improving the healthcare system that is conducive to public health” (*r* = 0.718) and “I can engage in political activities in collaboration with workers in other occupations to improve public health” (*r* = 0.703). However, the extent of exceeding the standard was nonsignificant, and the CVI values were evaluated as essential items at 0.92 and 1.00, respectively, in the results of the second content validity test by experts and were maintained. The correlation coefficient *r* = 0.344–0.679 for the remaining items, except for the above three items, confirmed the internal consistency of the items. The average alpha if an item was deleted to identify items that threaten reliability was 0.937, and all items were within the appropriate range of 0.935–0.940 (Supporting Table A9).

#### 3.2.2. Construct Validity: EFA and Naming Factors

EFA was conducted after the item-total correlation test to identify the construct validity of the 38 items. KMO and Bartlett's test of sphericity results for the 38 items were KMO = 0.900, and Bartlett's sphericity test value was also significant (*χ*^2^ = 5038.197, *p* < 0.001), suitable for EFA. As a result of the EFA, eight factors according to Kaiser's criterion [44] with an eigenvalue of one or more and the number of factors immediately before the slope of the scree plot started to level off and appeared somewhat ambiguous (*n* = 4–8; Supporting Table A10 and Supporting Figure A2).

Parallel analysis was performed to supplement this, and the number of factors for which the eigenvalues analyzed from the actual data were greater than the eigenvalues of the randomly generated data was four (Supporting Table A11). Therefore, extracting the four factors based on the results of Kaiser's criterion, a scree plot, and parallel analysis was identified as the most appropriate.

Accordingly, EFA was re-executed for 38 items by designating four factors, and then, after deleting three items with a factor loading of less than 0.3, an EFA was conducted again for 35 items, and the factor loading range was 0.344–0.747, with all items being 0.3 or higher. The results of the KMO and Bartlett's sphericity test were KMO = 0.905, *χ*^2^ = 4734.529, and *p* < 0.001, which was suitable for EFA. The cumulative explanatory power of the four factors was 55.483%, which satisfied the recommended standard explanatory power in the psychosocial science. All eigenvalues for each factor were above 1, and the cumulative factor for each item within the factor ranged from 0.493 to 0.809. For each factor, the number of items with a cumulative factor of 0.50 or more was also five or more, which satisfied the criteria of Costello and Osborne [43]. Therefore, four factors and 35 items were finalized. The four factors extracted through EFA were named by reflecting political knowledge, political efficacy, political interaction, and political activity during the concept development process by Han and Kim [1] (Table 1, Supporting Figure A3).

#### 3.2.3. Construct Validity: Convergent and Discriminative Validity

Through multitrait/multi-item matrix analysis, convergent validity showed that the correlation coefficient of each factor was *r* = 0.459–*r* = 0.752, and all 35 items were *r* = 0.40 or higher, meeting the test criteria. Discriminant validity was greater than the standard error value of two times for all 35 items; therefore, it met the test criteria (Supporting Table A12).

#### 3.2.4. Internal Consistency Reliability and Correlation Among Factors

The result of calculating the Cronbach's *α* across all items to test the internal consistency reliability was 0.940, and the Cronbach's *α* for each of the four factors ranged from 0.856 to 0.907, meeting the test standard that the newly developed scale should be 0.70 or higher [45] (Supporting Table A13).

The results of the correlation analysis among the factors of the tool showed a significant and relatively high correlation: political activity and political knowledge, *r* = 0.633 (*p* < 0.001); political activity and political interaction, *r* = 0.549 (*p* < 0.001); political efficacy and political activity, *r* = 0.541 (*p* < 0.001); and political efficacy and political knowledge, *r* = 0.506 (*p* < 0.001). A significant relatively low correlation was shown with political efficacy and political interaction *r* = 0.208 (*p*=0.002; Supporting Table A14).

### 3.3. Secondary Tool Test (Second Survey): Confirmed 35 Items Across Four Factors

As the reliability and validity of the tool comprising four factors and 35 items were established, CFA, criterion validity, and internal consistency reliability were analyzed to test the model fit of the Political Competence Scale for Nurses (PCS-N).

#### 3.3.1. Tool's Descriptive Statistics and CFA

**Figure 2:**
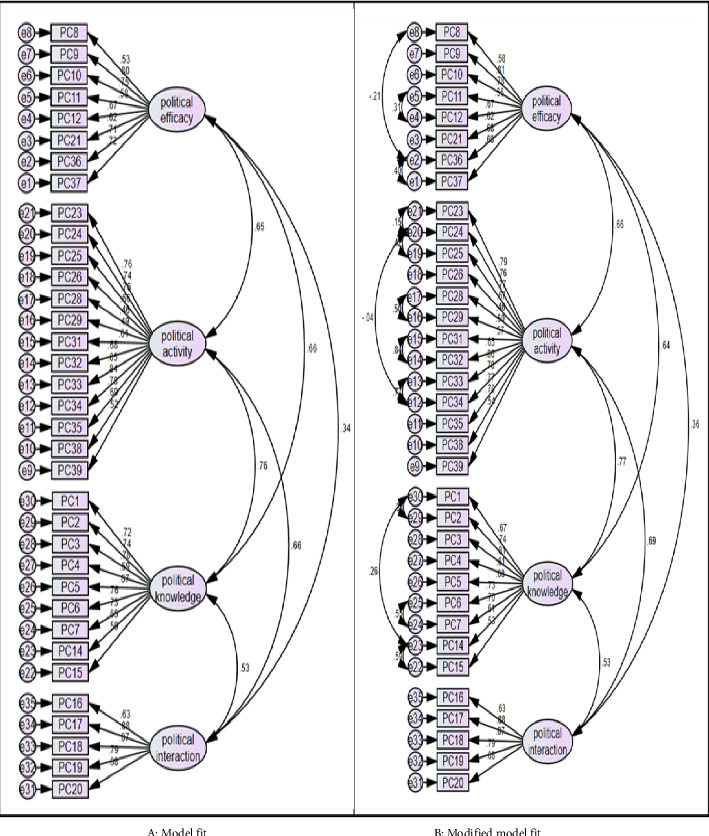
The proposed models of the tool by confirmatory factor analysis. (a) Model fit. (b) Modified model fit.

The average value of the PCS-N was 2.91 out of five points. By subfactor, political efficacy had the highest average (3.76 points), followed by political knowledge (2.87 points), political activity (2.71 points), and political interaction (1.87 points). The skewness value of political interaction was the highest (0.247), but all did not exceed ± 1.965, and it was normally distributed at a significance level of 0.05. To evaluate the suitability of the four-factor model of the established PCS-N, the CFA results showed that the standardized coefficient for evaluating concept validity was over 0.50, which is the general standard for 33 items or an approximation for two items, indicating that the items for each factor were valid. The chi-square test results were as follows: *p* < 0.001, *χ*^2^/df = 3.754, GFI = 0.644, RMR = 0.065, RMSEA = 0.111, CFI = 0.717, PGFI = 0.566, and PNFI = 0.608. The RMR of the absolute fit index and PNFI of the simple fit index met the criteria. Although some criteria for model fit were met, CFA was re-executed by adding correlations to items with a high covariance modification index of 20 or higher, as recommended by Schmitt [50] to improve the model fit. All model fit indices improved in the modified four-factor model: *χ*^2^/df = 2.223, GFI = 0.762, RMR = 0.060, RMSEA = 0.074, CFI = 0.877, PGFI = 0.654, and PNFI = 0.727. These met the criteria or indicated a model fit with values close to the criteria (Figure 2 and Table 2).

#### 3.3.2. Criterion Validity: Concurrent Validity and Known-Groups Validity

The result of the concurrent validity test, PCS-N, showed a significant correlation (*r* = 0.511, *p* < 0.001) with the political efficacy and political interest [28, 47] scales. In addition, there was a significant correlation with all subfactors' political efficacy (*r* = 0.503, *p* < 0.001), political activity (*r* = 0.468, *p* < 0.001), political knowledge (*r* = 0.431, *p* < 0.001), and political interaction (*r* = 0.261, *p* < 0.001); thus, concurrent validity was established (Table 3).

For the known-group validity analysis (*p* < 0.05), the group that had currently or had previously joined a political party had higher political competence than the nonmember group (*F* = 3.36, *p*=0.036). In addition, the group that was currently or had previously been a member of the Korea Nursing Association had higher levels than the nonmember group (*F* = 6.721, *p* < 0.001) and the group that was currently or had previously been a member of a volunteer group performed better than the nonmember group (*F* = 11.078, *p* < 0.001). The group that had presently or had previously joined a social group had significantly higher political competence than the group that did not (*F* = 11.094, *p* < 0.001) (Supporting Table A15).

#### 3.3.3. Internal Consistency Reliability and Correlation Among Factors

Cronbach's *α* for the 35 items was 0.951, similar to the primary test reliability of 0.940. For each factor, political efficacy was 0.868, political knowledge was 0.918, political activity was 0.889, and political interaction was 0.865, indicating excellent internal consistency (Table 4).

The correlations among factors in the PCS-N were as follows: political activity and political knowledge, *r = *0.698 (*p* < 0.001); political activity and political interaction, *r* = 0.647 (*p* < 0.001); political efficacy and political knowledge, *r* = 0.607 (*p* < 0.001); political efficacy and political activity, *r* = 0.576 (*p* < 0.001); and political efficacy and political interaction, *r* = 0.364 (*p* < 0.001) (Table 5).

### 3.4. Test–Retest Reliability (Third Survey) and Final PCS-N

The test–retest correlation coefficient of the PCS-N was 0.866 (*p* < 0.001), the correlation coefficient was over 0.70, and test–retest reliability was established, confirming the stability of the PCS-N. The PCS-N is presented in Supporting Information B.

## 4. Discussion

This study developed the PCS-N to measure nurses' political competence and established content validity, construct validity, criterion validity, and reliability. Specifically, as an initial phase to ensure that all factors of Han and Kim's [1] proposed concept of nurses' political competence were accurately reflected in the measurement tool items, expert content validity was tested, through which the CVI was calculated and the appropriateness of each item was evaluated, leading to the revision or removal of unnecessary items. After verifying content validity, construct validity was evaluated to determine whether the tool accurately measures the intended concept. This evaluation included item-total correlations, EFA, parallel analysis, convergent validity, discriminant validity, and CFA to determine whether the items were classified correctly according to the theoretically expected factor structure. Criterion validity was examined through tests of concurrent validity and known-group validity. Finally, the internal consistency and test–retest reliability of the tool were established.

The PCS-N comprised 35 items across four factors: political knowledge, political efficacy, political interaction, and political activity. Political knowledge comprised 9 items (PC1–PC9), political efficacy comprised 8 items (PC10–PC17), political interaction comprised 5 items (PC18–PC22), and political activity comprised 13 items (PC23–PC35). The PCS-N has a five-point (1–5) Likert scale and does not include reverse items. For example, political competence is calculated by summing the response scores and is scored as 1 = *strongly disagree*, 2 = *disagree*, 3 = *neutral*, 4 = *agree*, and 5 = *strongly agree*. Therefore, by summing the response scores of all items of the PCS-N, nurses' political competence can be determined, and the competence for each factor can also be identified by adding up the separate response scores for each of the four factors. The total scores range from 35 to 175 points (political knowledge ranges 9–45, political efficacy ranges 8–40, political interaction ranges 5–25, and political activity ranges 13–65 points).

This study is important because it is the first to develop an instrument that establishes validity and reliability in the absence of a tool for measuring nurses' political competence. In this regard, in political science, single-dimensional political efficacy [27–29], political interest [30, 31], political trust [28], political cynicism [51, 52], political attitude [53], political knowledge [54], political sophistication [55, 56], political skill [57], social participation capacity [58, 59], social justice advocacy capacity [60], and political participation [61] are used, but most of the tools are composed of a single dimension. The strength of the PCS-N is that it comprises multiple dimensions, including the field of political science, while reflecting the specificity of the nursing profession. Furthermore, Clark's [32] PAI can only measure nurses' political knowledge, political interaction, and political activities, while the PCS-N can also measure political efficacy.

The content validity of this study was verified by including professors at nursing colleges and political nurse activists who are well known for their rich experience in healthcare policy reform and successful political activities. Content validity is a different validity testing process than construct validity. It evaluates whether the measurement tool sufficiently reflects the concept it intends to measure and is mainly verified using CVI by an expert panel. Han and Kim's [1] concept of nurses' political competence and the vivid political experiences of activists with powerful political competence were well reflected in all items of the PCS-N.

To validate construct validity, the PCS-N was additionally subjected to O'connor's [41] and Henson and Roberts' parallel analyses [42], a precise factor extraction method, to minimize subjective intervention in the interpretation of slope changes in the EFA scree plots. In addition, as a result of using multitrait/multi-item matrix analysis, all items had a correlation coefficient of 0.459–0.752 with subscales excluding each item, and convergent validity was established by meeting the standard of at least 0.400. Discriminant validity was also established because of the low correlations discriminating with factors other than the relevant factor [44]. This indicates that the developed PCS-N has a high correlation among items constituting the subscales and simultaneously has discriminative power over items constituting other subscales, thereby measuring the unique properties of each subscale. Convergent and discriminant validity are other strengths of the PCS-N because they are known validation methods that significantly affect the acceptability of the tool. In addition, this study analyzed data collected in general environments before the outbreak of the COVID-19 pandemic rather than data that could have been affected by environmental factors related to the COVID-19 pandemic; thus, the generalizability of the results is somewhat advantageous. In the CFA results, which applied the modified four-factor model to evaluate the goodness-of-fit of the factor structure of the confirmed tool, the model fit of the PCS-N was suitable because the overall standard was satisfied or close to the standard value.

The criterion validity test used a concurrent validity test and a known-group validity test, which can evaluate the relevance of the measurement tool developed in this study compared with existing tools with proven reliability and validity or existing objective criteria. The concurrent validity test of criterion validity revealed that “political efficacy” [28, 62] and “political interest” [47, 62] showed a significant positive correlation with *r* = 0.511, and concurrent validity was established. This indicates that the PCS-N includes the specificity of measuring nurses' political competence and the attributes of political competence possessed by ordinary people. The results of the known-group validity test confirmed that political competence was significantly higher among those who had more past or current activities in political parties, representative nursing organizations, volunteer organizations, and civil society organizations, which are variables that influence political participation in previous studies [18, 63–66], and the known-group validity was consistent with previous studies. In this study, known-group validity was assessed, in addition to concurrent validity, which has been commonly used as a criterion validity test method in previous studies. This was because there were no tools to measure nurses' political competence multidimensionally, and there were not many multidimensional tools in politics. Therefore, it was not easy to find the exact correlation with simultaneous validity. The known-group validity test is meaningful because it is not yet widely used in nursing, and it is necessary to use various criterion validity tests in future tool development studies.

Cronbach's *α* of the first overall tool was 0.940 (0.856–0.907 by factor) and 0.951 (0.865–0.918 by factor) by the second round. Therefore, the newly developed tool's Cronbach's *α* was higher than the criterion to meet 0.70 or higher [45]; thus, the homogeneity of the PCS-N was established. Internal consistency reliability is a test used to evaluate whether the items of a measurement tool consistently measure the same concept [45]. A high Cronbach's alpha coefficient indicates that the items of the tool consistently measure the same concept, which strengthens the reliability of the tool. Cronbach's *α* is most commonly used because of the advantages that can be derived from one measurement, but it is necessary to consider applying more diverse methods in the future. In addition, as a result of the test–retest reliability test, the correlation coefficient was 0.866 (*p* < 0.001), and the stability of this PCS-N was established by the criterion that a correlation coefficient of 0.70 or higher is acceptable as a stable tool [54]. For the test–retest reliability test, 50 consenting participants among participants from the second data collection were resurveyed two weeks after the first questionnaire was administered to analyze the Pearson correlation. It is necessary to continuously evaluate the stability of the PCS-N by increasing the number of participants in the future.

This study is meaningful because the PCS-N, which is a valid and reliable measurement instrument, was developed as a multidimensional measure of nurses' political competence. Therefore, this instrument can measure both nurses' political efficacy and their ability to use political knowledge and information to perform political activities through political interaction to influence the government's policy decision-making process. The PCS-N can be used to objectively evaluate nurses' political competence. Based on prior results, the lack of formal and nonformal political education is reported to be the most notable barrier to political participation among nurses [2, 7, 8, 10]. Therefore, the PCS-N could be used as basic data for developing political education and training programs that could increase the political competence of nurses in undergraduate and graduate schools and in continuing education at representative nursing organizations in each country. Political competence enhanced through the application of the developed programs could contribute to the activation of nurses' political participation or healthcare policy intervention to advocate for public health rights, as well as to the expansion of the nursing workforce and improvement of working environments in nursing practice. In addition, this study could be used as research data to compare and evaluate the effectiveness of formal and informal political education programs for nursing students and nurses. Accumulating empirical data on nurses' political competence could provide a foundation for establishing a theoretical nursing model to strengthen political competence in the future.

Based on these results, the study limitations and suggestions for future research are as follows. First, the nonprobability convenience sampling method used to test the validity and reliability of the tool does not reflect the national distribution of South Korean nurses. Accordingly, there may have been problems with selection bias and generalization of the population. Therefore, it will be necessary to collect data from nurses nationwide or globally through probability sampling. Second, in the EFA, the cross-loaded items across the factors were not deleted because they were judged to be essential. However, it is necessary to delete and analyze the cross-loaded items in the future. Third, in the known-group validity test of criterion validity, group comparisons were made according to whether participants were active in political parties, nursing political organizations, volunteer groups, or civil society organizations, which were variables influencing political participation in previous studies. In the future, it will be necessary to analyze the factors that influence political competence by adding demographic and social characteristics such as sex, education level, income level, and religion. Fourth, in the test–retest reliability test, the correlation was analyzed with 50 participants; however, it will be necessary to test the stability of the tool by expanding the number of participants. Fifth, future research on the hierarchy among the factors of PCS-N—political knowledge, political efficacy, political interaction, and political activity—is needed.

## 5. Conclusions

In this study, a standardized PCS-N measurement tool comprising 35 items across four factors was developed through the following examination stages: content validity, construct validity, convergent validity, discriminant validity, criterion validity, internal consistency reliability, concept validity, model fit, and test–retest reliability of the political competence measurement items. The PCS-N measurement instrument can be used to evaluate nurses' political competence levels, provide a basis for constructing education and training programs to strengthen their political competence, and evaluate their effectiveness. First, nurses' political competence levels were identified using a measurement instrument with verified validity and reliability. Second, measuring nurses' political competence could provide basic data for education to strengthen their political competence, program development, and effect analysis.

## 6. Implication for Nursing Management

In most countries, nurses' political competence is insufficient owing to a lack of political participation or healthcare policy intervention. The PCS-N, which measures nurses' political competence, demonstrated the validity, reliability, and goodness-of-fit of the model in this study. Thus, the PCS-N measurement tool could be used to evaluate nurses' political competence levels and provide a basis for constructing education and training programs for political empowerment and effectiveness evaluation. In addition, representative nursing organizations can develop and apply different strategies to strengthen nurses' political competence according to their level. Through this, it will be possible to strengthen the political competence of nurses, promote political participation and intervention in healthcare policies, and advocate for the public right to health and the rights of the nursing profession.

## Figures and Tables

**Table 1:** Factor loading from exploratory factor analysis for PCS-N (35 items) (*N* = 224).

Items	Communalities	Factor I	Factor II	Factor III	Factor IV	Corrected item-total correlations	Alpha if item deleted	Alpha if item deleted within factor
9	0.62	**0.763**	0.124	0.152	0.005	0.504	0.939	0.835
36	0.552	**0.715**	0.145	0.14	−0.011	0.475	0.939	0.846
10	0.566	**0.714**	0.142	0.145	0.123	0.531	0.939	0.841
12	0.503	**0.678**	−0.044	0.203	0.023	0.402	0.94	0.852
11	0.451	**0.649**	0.088	0.125	−0.074	0.392	0.94	0.854
37	0.625	**0.637**	0.43	0.143	0.119	0.656	0.937	0.848
8	0.448	**0.629**	0	0.187	0.128	0.434	0.939	0.854
21	0.361	**0.583**	0.094	0.085	0.073	0.394	0.94	0.86
28	0.46	−0.023	**0.637**	0.152	0.177	0.474	0.939	0.904
39	0.517	−0.064	**0.635**	0.13	0.304	0.479	0.939	0.902
33	0.657	0.434	**0.633**	0.199	0.168	0.72	0.937	0.895
25	0.618	0.21	**0.624**	0.292	0.315	0.702	0.937	0.895
35	0.611	0.386	**0.612**	0.243	0.169	0.706	0.937	0.895
32	0.654	0.53	**0.598**	0.074	−0.102	0.582	0.938	0.9
34	0.604	0.44	**0.598**	0.16	0.166	0.682	0.937	0.897
24	0.634	0.043	**0.595**	0.415	0.324	0.661	0.937	0.897
36	0.524	0.174	**0.542**	0.428	0.13	0.631	0.938	0.9
39	0.434	−0.173	**0.537**	0.218	0.26	0.405	0.94	0.906
31	0.493	0.431	**0.536**	0.058	−0.129	0.476	0.939	0.904
23	0.508	0.291	**0.523**	0.218	0.321	0.651	0.937	0.899
38	0.498	0.426	**0.493**	0.099	0.252	0.613	0.938	0.9
3	0.651	0.089	0.169	**0.755**	0.212	0.569	0.938	0.862
4	0.584	0.136	0.153	**0.735**	0.046	0.512	0.939	0.854
2	0.624	0.266	0.057	**0.71**	0.214	0.576	0.938	0.854
6	0.607	0.01	0.356	**0.662**	0.202	0.588	0.938	0.857
7	0.523	0.051	0.277	**0.645**	0.166	0.539	0.938	0.874
1	0.496	0.373	0.059	**0.589**	0.079	0.524	0.939	0.859
15	0.45	0.259	0.239	**0.549**	0.158	0.575	0.938	0.863
5	0.344	0.236	0.099	**0.525**	0.05	0.432	0.939	0.859
14	0.546	0.438	0.29	**0.516**	0.067	0.647	0.938	0.863
17	0.747	−0.051	0.209	0.215	**0.809**	0.485	0.939	0.866
19	0.702	0.021	0.201	0.143	**0.801**	0.477	0.939	0.808
18	0.687	0.044	0.287	0.151	**0.762**	0.526	0.939	0.817
20	0.593	0.021	0.298	0.137	**0.696**	0.489	0.939	0.812
16	0.525	0.293	0.046	0.184	**0.635**	0.479	0.939	0.831

Eigenvalue		5.644	5.36	4.704	3.711			

% of variance		16.125	15.316	13.44	10.603			

Cumulative (%)		16.125	31.44	44.88	55.483			

*Note:* Factor I, political efficacy; Factor II, political activity; Factor III, political knowledge; Factor IV, political interaction. Each row in the table represents one item, and the factor loadings of each item are presented for four factors (Factor I to Factor IV). The factor column with the highest loading value for each item is indicated in bold.

Abbreviation: PCS-N, Political Competence Scale for Nurses.

**Table 2:** Standardized regression estimates of items and model fit of CFA (*N* = 225).

**Standardized regression estimates of items by factors**									
**Factors**	**Items**		**Estimate**	**S.E.**		**Standardized regression estimate**		**C.R.**	**p**

Political efficacy	PC37		1.000			0.681			
	PC36		1.020	0.086		0.681		11.817	< 0.001
	PC21		0.708	0.085		0.615		9.291	< 0.001
	PC12		0.952	0.107		0.667		8.909	< 0.001
	PC11		0.710	0.094		0.556		7.531	< 0.001
	PC10		0.984	0.097		0.776		10.165	< 0.001
	PC9		1.066	0.101		0.812		10.539	< 0.001
	PC8		0.772	0.099		0.575		7.766	< 0.001

Political activity	PC39		1.000			0.536			
	PC38		1.177	0.154		0.696		7.619	< 0.001
	PC35		1.377	0.171		0.767		8.039	< 0.001
	PC34		1.424	0.175		0.785		8.132	< 0.001
	PC33		1.330	0.162		0.796		8.194	< 0.001
	PC32		1.102	0.151		0.646		7.288	< 0.001
	PC31		1.005	0.150		0.570		6.722	< 0.001
	PC29		0.804	0.132		0.496		6.098	< 0.001
	PC28		0.847	0.146		0.465		5.812	< 0.001
	PC26		0.997	0.134		0.669		7.448	< 0.001
	PC25		1.359	0.169		0.767		8.036	< 0.001
	PC24		1.277	0.160		0.764		8.008	< 0.001
	PC23		1.329	0.163		0.786		8.138	< 0.001

Political knowledge	PC15		1.000			0.532			
	PC14		1.008	0.095		0.610		10.576	< 0.001
	PC7		1.174	0.159		0.701		7.404	< 0.001
	PC6		1.192	0.157		0.732		7.584	< 0.001
	PC5		1.307	0.179		0.682		7.300	< 0.001
	PC4		1.039	0.152		0.608		6.816	< 0.001
	PC3		1.370	0.172		0.810		7.986	< 0.001
	PC2		1.199	0.157		0.739		7.622	< 0.001
	PC1		1.035	0.143		0.675		7.241	< 0.001

Political interaction	PC20		1.000			0.679			
	PC19		0.918	0.087		0.786		10.567	< 0.001
	PC18		1.072	0.093		0.867		11.464	< 0.001
	PC17		1.041	0.090		0.881		11.601	< 0.001
	PC16		1.016	0.117		0.633		8.702	< 0.001

**Model fit of confirmatory factor analysis (CFA)**									
**Classification**		**Criterion**			**Factor model**		**Modified factor model**		

*x* ^2^		(*p* > 0.05)			2079.801 (*p* < 0.001)		1202.558 (*p* < 0.001)		
*x* ^2^ d.f.		≤ 3			3.754		2.223		
GFI		≥ 0.80			0.644		0.762		
RMR		0.05–0.08			0.065		0.06		
RMSEA		≤ 0.08			0.111		0.074		
CFI		≥ 0.9			0.717		0.877		
PGFI		0.6–0.09			0.566		0.654		
PNFI		0.6–0.09			0.608		0.727		

Abbreviations: CFI, comparative fit index; GFI, goodness-of-fit index; PGFI, parsimonious goodness-of-fit index; PNFI, parsimonious normed fit index; RMR, root mean residual; RMSEA, root mean square error of approximation.

**Table 3:** Concurrent validity of Political Competence Scale for Nurses (PCS-N) (*N* = 225).

Factors	Concurrent validity
	Political efficacy and interest: *r* (*p*)
Political efficacy	0.503 (< 0.001)
Political activity	0.468 (< 0.001)
Political knowledge	0.431 (< 0.001)
Political interaction	0.261 (< 0.001)
Total	0.511 (< 0.001)

*Note:* Concurrent validity—among PCS-N factors and the tool of political efficacy and interest (Craig et al. [28] and Banducci and Semetko [47]).

**Table 4:** Internal consistency reliability of PCS-N (*N* = 225).

Factors	Reliability	
	Number of items	Cronbach's *α*
Political efficacy	8	0.868
Political activity	13	0.918
Political knowledge	9	0.889
Political interaction	5	0.865
Total	35	0.951

**Table 5:** Correlations among factors of PCS-N (*N* = 225).

Factors	Statistics			Correlations among factors: *r* (*p*)			
	Mean ± SD	Skewness	Kurtosis	Political efficacy	Political activity	Political knowledge	Political interaction
Political efficacy	3.76 ± 0.55	−0.023	−0.651	1			
Political activity	2.71 ± 0.62	0.028	0.450	0.576 (< 0.001)	1		
Political knowledge	2.87 ± 0.61	0.054	0.302	0.607 (< 0.001)	0.698 (< 0.001)	1	
Political interaction	1.87 ± 0.66	0.247	−0.344	0.364 (< 0.001)	0.647 (< 0.001)	0.478 (< 0.001)	1
Total	2.91 ± 0.51	0.108	0.266	—	—	—	—

*Note:* Interitem correlation coefficients among all PCS-N factors: Pearson correlation coefficients.

## Data Availability

The data that support the findings of this study are available in the Supporting Information of this article.

## Supporting Information

Supporting Information include two files that provide additional information related to this article.

Supporting Information 1 Supporting Information A contains about conceptual framework of nurses' political competence measurement scale, participants' selection criteria and demographic characteristics for test the reliability and validity test of scale, result of content validity, item-total correlations of preliminary items, exploratory factor analysis, parallel analysis, multitrait/multi-item matrix, correlations among factors of political competence scale for nurses, and verification of known-groups validity.

Supporting Information 2 Supporting Information B includes the Political Competence Scale for Nurses (PCS-N) items verified for reliability, validity, and model fit.
