# Supplementary material for: The Political Competence Scale for Nurses (PCS-N): Instrument Development and Psychometric Evaluation
Source: J Nurs Manag. 2025 May 19;2025:4683994. doi: 10.1155/jonm/4683994 (PMC12105907; doi:10.1155/jonm/4683994)
Supplement: Supporting Information 1 — Supporting Information A contains about conceptual framework of nurses' political competence measurement scale, participants' selection criteria and demographic characteristics for test the reliability and validity test of scale, result of content validity, item-total correlations of preliminary items, exploratory factor analysis, parallel analysis, multitrait/multi-item matrix, correlations among factors of political competence scale for nurses, and verification of known-groups validity. [file 4683994.f1.docx]

**Supplementary material A.**

**Conceptual framework of this research (Han & Kim, 2020)**

In this study, the concepts and indicators of Han & Kim (2020), who presented a multidimensional conceptualization of nurses' political competence, were used as the conceptual framework for developing a measure of nurses' political competence using Hybrid model (Schwartz-Barcott & Kim, 2000).

It consists of four factors and 15 attributes and indicators: Political Knowledge, Political Efficacy, Political Interaction, and Political Activity. The attributes of each dimension include: Political Knowledge: political information, political knowledge, and systematic analysis ability; Political Efficacy: internal political efficacy, external political efficacy, and self-pride of nursing profession; Political Interaction: group and volunteer activities, networking, and persuasion; and Political Activity: political leadership, political expression, assertive action, political advocacy, political participation, and policy intervention.

Specifically, the ‘political information’ attribute under the ‘Political Knowledge’ dimension implies ‘collecting information related to social changes and healthcare policy from various sources such as TV, newspapers, and social network service, confirming and analyzing political information, and utilizing political information through media,’ the ‘political knowledge’ means ‘having knowledge centered on current events or specific issues related to governments, political system, policy-making process, and various political actions occurring in the current society,’ and ‘systematic analysis ability’ refers ‘investigating and analyzing social phenomena and healthcare issues with insight, clarifying political issues, and making political judgments and inferences by reflecting the latest trends.’

The ‘internal political efficacy’ attribute under the ‘Political Efficacy’ dimension means ‘the degree to which one feels one can understand the political process as a citizen, the degree of belief in politics and social change, and the expectation or belief that one can exercise political influence,’ ‘external political efficacy’ implies ‘having expectations or beliefs that the government authorities and institutions will respond to citizens’ demands, having political trust, which is a positive expectation of others’ intentions or actions, and having political interest, which is an interest in political issues or politics in general,’ and ‘pride in the nursing profession’ connotes ‘having a sense of responsibility and professional ethics as a professional to implement the nursing responsibility and having a clear nursing professional perspective.

The ‘organizations and service activities’ attribute under the dimension of ‘Political Interaction’ means ‘having common experiences and a sense of belonging among members within a professional representative organization, and performing group activities that go beyond nursing through joining civil society groups or doing community service,’ ‘networking’ means ‘forming effective relationships beyond nursing through partnerships with nursing recipients, outsiders, public and private related organizations, and groups, and building a network,’ and ‘persuasive power’ means ‘recognizing political differences and diversity, understanding opposing positions, listening with patience, dealing with conflicts, compromising and coexisting through discussion and consultation, achieving desired goals, contacting the government, members of the National Assembly, the public, the media, and engaging in activities such as communication, lobbying, and forming public opinion.’

The ‘political expression’ attribute under the dimension of ‘Political Activity’ means ‘expressing the political views or content you want to convey in logical words or letters,’ ‘assertive action’ refers to ‘raising issues regarding nurses’ interests and rights autonomously and proactively,’ ‘political leadership’ implies ‘having a vision under a strategic perspective, being dedicated to the organization, giving trust to others, having empathy and inclusiveness, and being expandable through horizontal relationships,’ ‘political advocacy’ means ‘recognizing and performing the role of a spokesperson who improves the public's health rights and health care environment or contributes to society while having a sense of social responsibility as a healthcare professional,’ ‘political participation’ refers ‘participating in political processes and activities as a citizen through voting, elections, and using internet media, and reflecting citizen-centered health policy changes and nursing perspectives,’ and ‘policy intervention’ implies ‘recognizing the health care environment and social issues, understanding the legislative and policy decision-making processes, and influencing policymaking’.

**
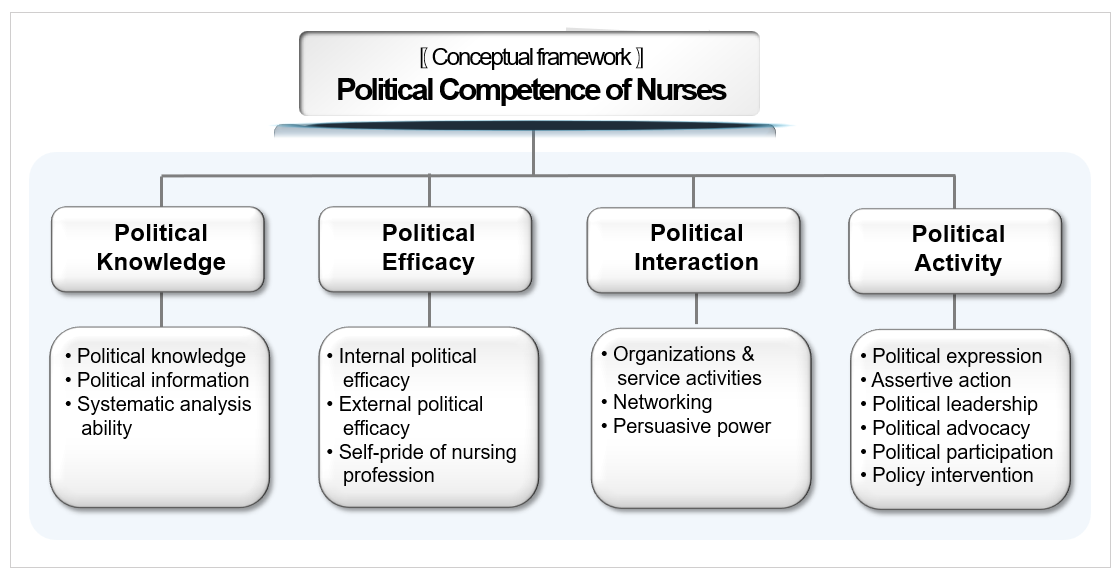
**

**Figure A1.** Conceptual framework: Concept of Political Competence for nurses (Han & Kim, 2020)

**Table A1.** Concept of the Political Competence for Nurses (Han & Kim, 2020)

| Dimension | Attributes | Indicators (Theoretical/ Empirical) [Sources] | Explanation |
| --- | --- | --- | --- |
| Political  knowledge  Political  knowledge | Political knowledge | ∙Having structural and factual knowledge related to the political system, political process, specific issue, and etc [1-3,10,11,15,20] | Having knowledge of political system, process, specific issue, and health care policy |
|  |  | ∙Having professional knowledge about the state or local government, political system, political process, health care policy |  |
|  | Political information | ∙Collecting and utilizing political information through various sources [10,13] | Collecting and utilizing of political information on various media |
|  |  | ∙Collecting and utilizing political issues and information related to health care policy through various media |  |
|  | Systematic analysis ability | ∙Systematically analyzing political events, problems, and decisions [2,3,11,13]  ∙Reflecting the latest trends, political judgment and reasoning [1-3,10,11,15] | Systematically analyzing healthcare data and suggesting reasonable alternatives |
|  |  | ∙Collecting data related to health care and analyzing it by critical thinking  ∙Providing a reasonable alternative to improve health care policy |  |
| Political  efficacy | Internal  political efficacy | ∙Participating effectively in politics and recognizing that individuals can understand the political process [19,5-7,9,18,19]  ∙The expectation or belief in being able to exert political influence [7-9,14,15,17,19] | Having faith in the exercise of political influence and political achievement |
|  |  | ∙Having political self-concepts and self-assurance as citizens  ∙Having a political consciousness and a belief system for change |  |
|  | External  political efficacy  Self-pride of nursing profession | ∙The expectation or belief that government authorities and agencies will respond to the needs of citizens [7-9]  ∙Being interested in political issues [5,7,9,15,19]  ∙Interested in health care policies related to public and nurses [23] | Having confidence in the government or local governments and being interested in political issues or health care policies  Recognizing political  participation in  improving national  health based on the |
|  |  | ∙Being interested in social issues or political issues as a basic motivation for political participation or use of news media |  |
|  |  | ∙Having pride in nursing profession  ∙Belief in political achievement through solidarity of nursing profession  ∙Improvement of the health care system is a duty owed in terms of professional ethics | pride of nursing profession |
| Political  interaction  Political  interaction | Organizations and service activities | ∙Having a common experience among members within a political community [1-3,9,13,21]  ∙Having a sense of belonging within a political community [1-3,21]  ∙Participating in heterogeneous groups or community service [1-4,12] | Participating in activities of professional, civil society organizations or performing activities of community service |
|  |  | ∙Participation in nursing and health-related nursing organizations in interest  ∙Activating by joining civil society organizations, etc.  ∙Carrying out collective activities, such as community service, beyond nursing |  |
|  | Networking | ∙Establishing and maintaining a cooperative relationship of disparate groups or related political or social institutions [3,9,12] | Establishing cooperative relationships and activities with external organizations to improve the health care system |
|  |  | ∙Establishing cooperative relationships with government and political institutions  ∙Maintaining close relationships of the community and external organizations |  |
|  | Persuasive power | ∙Listening the other's opinions and requests constructively [2,3,13]  ∙To compromise and cooperate through discussion, consultation, etc [2,3,9,13]  ∙Acknowledging political differences and diversity, understanding opposition positions with patience and coordinating conflicts [1,3,9,13,15]  ∙Persuading policy decision makers and lobbying effectively [1,13,15] | Recognizing diversity and differences, persuade decision makers with patience, and build public opinion to improve the health care system |
|  |  | ∙Respecting the diversity of the other's experiences and perceptions  ∙Persuading the other person through logical data and discussion  ∙Listening with patience, understanding opposition and dealing with conflict  ∙Persuading policy decision makers and lobbying effectively  ∙Activities such as contacting the government, lawmakers, the public, and the media and forming public opinion |  |
| Political activity  Political activity | Political expression | ∙Expressing political ideas or opinions in words  [14-16]  ∙Representing political ideas or opinions with documents [14-16] | Expressing political views easily in words or writings |
|  |  | ∙Expressing in a coherent and logical language the views or content to convey  ∙Expressing the opinions or contents to be conveyed in logical documents |  |
|  | Assertive action | ∙Assertive behavior that conveys the viewpoint of national health [15,21-24]  ∙Raising the issue of nurse's interests and rights [14,21,24] | Raising the rights of nurses and the improvement of working conditions, etc. |
|  |  | ∙Demanding the rights and rights of nurses to raise issues and improve working conditions |  |
|  | Political leadership | ∙Being confident of having a friendly and independent view and responsibility [2,7]  ∙Leading and structuring individuals or organizations in the right direction [3,7,14] | Facilitating interdisciplinary cooperation with strategic vision |
|  |  | ∙Having a strategic perspective and vision  ∙Prioritizing the interests in public and nursing professions  ∙Demonstrating the leadership of service (empathy, trust, inclusion, horizontal relationship, etc.) in interdisciplinary cooperation |  |
|  | Political advocacy | ∙Respecting the views of the people [2,3,14,22,24]  ∙Having a willingness to change the target-oriented healthcare environment [22,24] | Acting as advocates and improving the system for public health advocacy |
|  |  | ∙Thinking about the role of advocate for the health of the people  ∙Willingness to improve health care system |  |
| Political activity | Political participation | ∙Legitimate acts with the aim of influencing the election of government officials, government actions and public policy [2,3,7-9,12,14,15,19] | Participating in elections and engaging in political activities as a citizen |
|  |  | ∙Participating in political processes and activities such as voting, election, internet media use (online, offline) as citizens |  |
|  | Policy intervention | ∙Understanding the policy making process [9,12,23,24]  ∙Evidence-based research skills that influence policy decisions [1,12,15]  ∙Having policy development skills [1,9,12,15,23,24]  ∙Impact on policy formulation [2,9,12,15,21,23,24] | Knowing and participating in health care policy decision-making and legislative processes |

**List of articles in theoretical sources above (Table A1).**

1. Warner JR. A phenomenological approach to political competence: Stories of nurse activists. Policy, Politics, & Nursing Practice. 2003;4(2):135-143. https://doi.org/10.1177/1527154403004002007

2. O'Grady ET, Johnson J. Health policy issues in changing environments. In: Hamric AB, Hanson CM, Tracy MF, O'Grady ET, editors. Advanced Practice Nursing: An Integrative Approach. 5th ed. St. Louis (MO): Elsevier Saunders; 2014. p. 595-602.

3. Melo WSD, Oliveira PJFD, Monteiro FPM, Santos FCDA, Silva MJND, Calderon CJ, et al. Guide of attributes of the nurse's political competence: A methodological study. Revista Brasileira de Enfermagem. 2017;70(3):526-534. https://doi.org/10.1590/0034-7167-2016-0483

4. Primomo J, Björling EA. Changes in political astuteness following nurse legislative day. Policy, Politics, & Nursing Practice. 2013;14(2):97-108. https://doi.org/10.1177/1527154413485901

5. Almond GA, Verba S. The civic culture: Political attitudes and democracy in five nations. Princeton (NJ): Princeton University Press; 1963. p. 1-574.

6. Campbell A, Gurin G, Miller WE. The voter decides. Evanston (IL): Row, Peterson; 1954. p. 1-242.

7. Barnes SH. Participation, education, and political competence: Evidence from a sample of Italian socialists. American Political Science Review. 1966;60(2):348-353. https://doi.org/10.2307/1953361

8. Converse PE. Change in the American electorate. In: Campbell A, Converse PE, editors. The Human Meaning of Social Change. New York: Russell Sage Foundation; 1972. p. 263-337.

9. Fratczak-Rudnicka B, Torney-Purta J. Competencies for civic and political life in a democracy. In: Rychen DS, Salganik LH, McLaughlin ME, editors. Contributions to the Second DeSeCo Symposium. Proceedings of the 2nd International DeSeCo Symposium; 2002 Feb 11–13; Swiss Federal Statistical Office, Geneva, Switzerland. Neuchâtelp (Switzerland): Swiss Federal Statistical Office; 2003. p. 71-86.

10. Pausch M. Democratic citizenship as an indicator for the quality of democracy. Paper presented at: The 22nd IPSA World Congress of Political Science; 2012 Jul 8–12; Madrid, Spain.

11. Gabriel OW. Politische einstellungen und politische kultur. In: Gabriel OW, Kropp S, editors. Die EU-Staaten im Vergleich: Strukturen, Prozesse, Politikinhalte. 3rd ed. Wiesbaden: VS Verlag für Sozialwissenschaften; 2008. p. 181-214. German

12. Jansson BS. Becoming an effective policy advocate: From policy practice to social justice. 4th ed. Pacific Grove (CA): Brooks/Cole Publishing Company; 2003. p. 1-464.

13. Krouse R, Marcus G. Electoral studies and democratic theory reconsidered. Political Behavior. 1984;6(1):23-39. https://doi.org/10.1007/bf00988227

14. Krammer R. Kompetenzen durch politische bildung: Ein kompetenz-strukturmodell. Informationen zur Politischen Bildung. 2008;29:5-14. German

15. Detjen J, Massing P, Richter D, Weißeno G. Politikkompetenz – ein modell. Wiesbaden: Springer-Verlag; 2012. p. 35-64. https://doi.org/10.1007/978-3-658-00785-0

16. Easton D. The political system: An inquiry into the state of political science. Political Science Quarterly. 1953;68(3):434-436. https://doi.org/10.2307/2145610

17. National Institute of Korean Language. Standard Korean language dictionary [Internet]. Seoul: National Institute of Korean Language; c2008 [cited 2018 Jul 12]. Available from: https://stdict.korean.go.kr/main/main.do.

18. Craig SC, Niemi RG, Silver GE. Political efficacy and trust: A report on the NES pilot study items. Political Behavior. 1990;12(3):289-314. https://doi.org/10.1007/bf00992337

19. Abravanel MD, Busch RJ. Political competence, political trust, and the action orientations of university. The Journal of Politics. 1975;37(1):57-82. https://doi.org/10.2307/2128891

20. Vis JCPM. Citizenship and political knowledge. In: Csepeli G, German D, Kéri L, Stumpf I, editors. From Subject to Citizen. Budapest: Hungarian Center for Political Education; 1995. p. 33-54.

21. Primomo J. Changes in political astuteness after a health systems and policy course. Nurse Educator. 2007;32(6):260-264. https://doi.org/10.1097/01.NNE.0000299480.54506.44

22. Bu X, Jezewski MA. Developing a mid‐range theory of patient advocacy through concept analysis. Journal of Advanced Nursing. 2007;57(1):101-110. https://doi.org/10.1111/j.1365-2648.2006.04096.x

23. Deschaine JE, Schaffer MA. Strengthening the role of public health nurse leaders in policy development. Policy, Politics, & Nursing Practice. 2003;4(4):266-274. https://doi.org/10.1177/1527154403258308

24. Arabi A, Rafii F, Cheraghi MA, Ghiyasvandian S. Nurses' policy influence: A concept analysis. Iranian Journal of Nursing and Midwifery Research. 2014;19(3):315-322.

**Participants and Psychometric Tests: The results of the scale’s Reliability and Validity**

According to the criterion of Tinsley and Tinsley [41], the number of subjects for Validity, Reliability, and CFA of the items test should be five times or more, and at least 200 participants, 236 subjects were selected for the study considering thirty-nine items of the preliminary tool and the non-response rate of 10-20% in most previous studies.

First, the 1st survey data collection for Item-total Correlations, EFA, Convergent and Discriminant Validity, and Internal Consistency Reliability tests were conducted from September 13 to October 5, 2018, targeting 224 participants after excluding missing values and unqualified subjects. Subsequently, the tool's 2nd questionnaire data collection for the Criterion Validity, Internal Consistency Reliability, Concept Validity, and CFA test was conducted from October 29 to November 18, 2018, targeting 225 participants, excluding missing values. Lastly, the 3rd survey data collection for testing the Test-retest Reliability of the tool was conducted from November 12th to November 27th, 2018, 14 days after the survey was completed, targeting 50 participants who agreed to the survey among the 2nd survey subjects (Supplementary Table A2).

Table A2. Participants' selection criteria for the reliability and validity test of measurement scale

| Categories | Selection criteria | Study subject |
| --- | --- | --- |
| 1^st^ survey |  |  |
| - Construct validity  Item Analysis Exploratory factor analysis Convergent validity Discriminant validity  - Internal consistency reliability (1st) | - Tinsley & Tinsley (1987)  Sample size: At least five times the number of items, with a minimum of 200 participants  - 15% dropout rate | - 236 nurses  ∙ Metropolitan city (118): 79 from medical institutions (2-3 sites), 39 from healthcare organizations and educational institutions (4-5 sites)  ∙ provinces (118): 78 medical institutions, 40 healthcare organizations and educational institutions |
| 2^nd^ survey |  |  |
| - Criterion validity  - Confirmatory Factor Analysis  - Internal consistency reliability (2nd) | - Tinsley & Tinsley (1987)  Sample size: At least five times the number of items, with a minimum of 200 participants  - 15% dropout rate | - 236 nurses  ∙ Metropolitan city (118): 79 from medical institutions (2-3 sites), 39 from healthcare organizations and educational institutions (4-5 sites)  ∙ provinces (118): 78 medical institutions, 40 healthcare organizations and educational institutions |
| 3^rd^ survey |  |  |
| Test-retest reliability | Tinsley & Tinsley (1987) | - 50 nurses |

**Participants’ characteristics**

The general characteristics of the 1^st^ survey participants are as (Supplementary Table A2.2) and 2^nd^ survey participants are as (Supplementary Table A3).

Table A3. General characteristics of participants: Demographic (1^st^ survey) (N = 224)

| Characteristics | Categories | N (%) | Mean±SD |
| --- | --- | --- | --- |
| Age (yrs) | 22~29 | 57(25.4) | 37.16±8.30 |
|  | 30~39 | 79(35.3) |  |
|  | 40~49 | 65(29.0) |  |
|  | 50~59 | 23(10.3) |  |
| Gender | Female | 219(97.8) |  |
|  | Male | 5(2.2) |  |
| Marital status | Unmarried | 92(41.1) |  |
|  | Married | 131(58.5) |  |
|  | Etc. | 1(0.4) |  |
| Education | Diploma | 41(18.3) |  |
|  | Bachelor | 132(58.9) |  |
|  | Master | 44(19.6) |  |
|  | Doctoral | 2(0.9) |  |
|  | Etc. | 5(2.2) |  |
| Religion | Christianity | 52(23.2) |  |
|  | Catholic | 33(14.7) |  |
|  | Buddhism | 24(10.7) |  |
|  | None | 112(50.0) |  |
|  | Etc. | 3(1.3) |  |
| Self-reported income status | High | 2(0.9) |  |
|  | High-Middle | 28(12.5) |  |
|  | Middle | 148(66.1) |  |
|  | Middle-Lower | 38(17.0) |  |
|  | Lower | 8(3.6) |  |
| Residence | Metropolitan | 135(60.3) |  |
|  | Small & medium-sized | 86(38.4) |  |
|  | Rural | 3(1.3) |  |

Table A3. Continued

| Characteristics | Categories | N (%) | Mean±SD |
| --- | --- | --- | --- |
| Working area | Hospital | 146(65.2) |  |
|  | Community | 78(34.8) |  |
| Region | Metropolitan city | 117(52.2) |  |
|  | Province | 107(47.8) |  |
| Position | Staff nurse | 157(70.1) |  |
|  | Charge nurse | 30(13.4) |  |
|  | Head nurse and bove | 27(12.1) |  |
|  | Etc. | 10(4.5) |  |
| Career present institution  (yrs) | 3-5 | 82(36.6) | 9.82±7.43 |
|  | 6-10 | 68(30.4) |  |
|  | 11-15 | 27(12.1) |  |
|  | 16-20 | 23(10.3) |  |
|  | > 20 | 24(10.7) |  |
| Total career period  (yrs) | 3-5 | 50(22.3) | 11.88±7.69 |
|  | 6-10 | 66(29.5) |  |
|  | 11-15 | 46(20.5) |  |
|  | 16-20 | 28(12.5) |  |
|  | > 20 | 34(15.2) |  |
| Joining a party | At present | 11(4.9) |  |
|  | In the past but not currently joined | 23(10.3) |  |
|  | Never joined | 190(84.8) |  |
| Joining a union | At present | 167(74.6) |  |
|  | In the past but not currently joined | 16(7.1) |  |
|  | Never joined | 41(18.3) |  |
| Joining nursing organizations | At present | 29(12.9) |  |
|  | In the past  but not currently joined | 18(8.0) |  |
|  | Never joined | 17(79.0) |  |

Table A4. General characteristics of participants: Demographic (2^nd^ survey) (N = 225)

| Characteristics | Categories | N (%) | Mean±SD |
| --- | --- | --- | --- |
| Age (yrs) | 22~29 | 52(23.1) | 39.95±7.77 |
|  | 30~39 | 72(32.0) |  |
|  | 40~49 | 59(26.2) |  |
|  | 50~59 | 40(17.8) |  |
|  | above 60 | 2(0.9) |  |
| Gender | Female | 220(97.8) |  |
|  | Male | 5(2.2) |  |
| Marital status | Unmarried | 88(39.1) |  |
|  | Married | 135(60.0) |  |
|  | Etc. | 2(0.9) |  |
| Education | Diploma | 50(22.2) |  |
|  | Bachelor | 134(59.6) |  |
|  | Master | 38(16.9) |  |
|  | Doctoral | 1(0.4) |  |
|  | Etc. | 2(0.9) |  |
| Religion | Christianity | 58(25.8) |  |
|  | Catholic | 31(13.8) |  |
|  | Buddhism | 22(9.8) |  |
|  | None | 111(49.3) |  |
|  | Etc. | 3(1.3) |  |
| Self-reported income status | High | 1(0.4) |  |
|  | High-Middle | 19(8.4) |  |
|  | Middle | 154(68.4) |  |
|  | Middle-Lower | 46(20.4) |  |
|  | Lower | 5(2.2) |  |
| Residence | Metropolitan | 108 (48.0) |  |
|  | Small & medium-sized | 102 (45.3) |  |
|  | Rural | 15 (6.7) |  |
|  |  | 108 (48.0) |  |
|  |  | 102 (45.3) |  |
|  |  | 15 (6.7) |  |

Table A4. Continued

| Characteristics | Categories | N (%) | Mean±SD |
| --- | --- | --- | --- |
| Working area | Hospital | 154(68.4) |  |
|  | Community | 71(31.6) |  |
| Region | Metropolitan city | 109(44.1) |  |
|  | Province | 116(55.9) |  |
| Position | Staff nurse | 153(68.0) |  |
|  | Charge nurse | 29(12.9) |  |
|  | Head nurse and above | 28(12.4) |  |
|  | Etc. | 15(6.7) |  |
| Career present institution  (yrs) | 3-5 | 80(35.6) | 10.99±8.08 |
|  | 6-10 | 48(21.3) |  |
|  | 11-15 | 34(15.1) |  |
|  | 16-20 | 31(13.8) |  |
|  | > 20 | 32(14.2) |  |
| Total career period  (yrs) | 3-5 | 41(18.2) | 14.07±8.64 |
|  | 6-10 | 57(25.3) |  |
|  | 11-15 | 40(17.8) |  |
|  | 16-20 | 30(13.3) |  |
|  | > 20 | 57(25.3) |  |
| Joining a party | At present | 9(4.0) |  |
|  | In the past but not currently joined | 18(8.0) |  |
|  | Never joined | 198(88.0) |  |
| Joining a union | At present | 132(58.7) |  |
|  | In the past but not currently joined | 13(5.8) |  |
|  | Never joined | 80(35.6) |  |
| Joining nursing organizations | At present | 24(10.7) |  |
|  | In the past  but not currently joined | 13(5.8) |  |
|  | Never joined | 188(83.6) |  |
| Joining service groups | At present | 26(11.6) |  |
|  | In the past  but not currently joined | 23(10.2) |  |
|  | Never joined | 176(78.2) |  |
| Joining civic groups | At present | 1.8(1.8) |  |
|  | In the past  but not currently joined | 2.7(2.7) |  |
|  | Never joined | 95.6(95.6) |  |

**Content Validity and preliminary survey**

The content validity test was performed two times by a total of eight experts in the fields of nursing, psychology, healthcare policy and political activists.

The first content validity test was conducted on the initial 40 items. As a result of the 1st content validity test, the average value of the CVI index of all items was .88, and the items with an I-CVI index of less than .80 appeared as two items and 38 items were derived by deleting or modifying some of the items (Supplementary Table A5).

Table A5. Result of Content Validity (1st)

| No. | Items | CVI | Results |
| --- | --- | --- | --- |
| 1 | I have knowledge of political systems, political processes, and healthcare policies. | 0.90 | Revision |
| 2 | I continually strive to expand the breadth and depth of my knowledge related to politics. | 0.80 |  |
| 3 | I am aware of current political and social healthcare issues. | 0.85 | Revision |
| 4 | I gather information about political and social or healthcare issues through a variety of media (TV, daily newspaper, internet, social media, and nurses' newspaper. | 0.90 | Revision |
| 5 | I can systematically identify key health care issues. | 0.90 | Revision |
| 6 | I can formulate reasonable alternatives to key health care issues. | 0.75 | Revision |
| 7 | I can utilize political information from a variety of media sources. | 0.90 | Revision |
| 8 | I am proud to be a nurse. | 0.90 |  |
| 9 | I believe it is the duty of nurses to participate in politics to improve public health. | 0.95 |  |
| 10 | I feel qualified to participate in activities to improve the health care system. | 0.95 |  |
| 11 | I believe that my participation in elections and voting behavior can have an important impact on society. | 0.90 |  |
| 12 | I believe that nurses' solidarity will increase the likelihood of political achievement. | 0.95 |  |
| 13 | I believe that politicians and public officials are working to improve the lives of the public. | 0.75 | Revision |
| 14 | I am more concerned about social issues and politics than other people. | 0.85 |  |
| 15 | I enjoy talking about political issues with others more than others. | 0.80 |  |
| 16 | I have participated in the activities of a nursing professional organization. | 0.95 | Revision |
| 17 | I have contacted other organizations to improve the healthcare system. | 0.80 | Revision |
| 18 | I have collaborated with other organization to enhance networking. | 0.90 | Revision |
| 19 | I have been a member of a community or external organization. | 0.90 | Revision |
| 20 | I have participated in the activities of a civil society organization or performed social service activities. | 0.90 |  |
| 21 | I respect the diversity of experiences and perceptions of others. | 0.90 |  |
| 22 | I am able to listen patiently to others whose political views are opposite to mine. | 0.80 | Revision |
| 23 | I can convince coworkers to participate in political activities to improve the healthcare system. | 0.95 |  |
| 24 | I am able to meet with government and local policymakers to convince them to improve healthcare policies. | 0.95 |  |
| 25 | I can shape public opinion in favor of healthcare reform. | 0.85 | Revision |
| 26 | I can explain my political views in a coherent way in speech or writing. | 0.95 |  |
| 27 | I have participated in all elections in the last two years. | 0.80 | Revision |
| 28 | I have posted comments on sites or social media related to political events or social issues. | 0.85 |  |
| 29 | I have voluntarily worked for a political party or candidate. | 0.85 |  |
| 30 | I have voluntarily donated fund to a political party or candidate. | 0.85 |  |
| 31 | I am able to advocate for improved working conditions in my nursing practice. | 0.85 | Revision |
| 32 | I can advocate for an expanded role in nursing. | 0.85 | Revision |
| 33 | I have a vision for improving the healthcare system to benefit the health of the population. | 0.90 |  |
| 34 | I have a vision for the advancement of the nursing profession. | 0.90 |  |
| 35 | I am able to work collaboratively with others in other professions in political activities. | 0.85 | Revision |
| 36 | I believe it is important for nurses to be politically active to advocate for the public. | 0.95 |  |
| 37 | I am willing to work to improve the healthcare system to advocate for the public. | 0.90 |  |
| 38 | I am committed to solving problems that arise in nursing practice to protect the rights of the public. | 0.95 | Revision |
| 39 | I am familiar with the healthcare policy-making and legislative processes. | 0.80 | Revision |
| 40 | I have expressed my opinion on legislative and policy decisions to improve the health of the public. | 0.90 | Revision |

2nd content validity test for the revised 38 items, the average value of the CVI index of all items was .91, and the items with an I-CVI index of less than .80 were found as three items, and a preliminary tool of 39 items was finally confirmed through separation of some items (Supplementary Table A6).

Table A6. Result of content validity (2nd)

| No. | Items | CVI | Results |
| --- | --- | --- | --- |
| 1 | I know current political and social issues. | 1.00 |  |
| 2 | I know the recent main healthcare issues. | 1.00 |  |
| 3 | I know the healthcare policy-making and legislative processes in my country. | 0.92 | Separation of item |
| 4 | I gather information about major healthcare issues through various methods (TV, daily newspaper, internet, social media, nurses' newspaper. | 0.83 | Revision |
| 5 | I can systematically analyze information on key healthcare issues. | 0.92 | Revision |
| 6 | I can formulate reasonable alternatives to healthcare issues to improve public health. | 0.83 |  |
| 7 | I am proud to be a nurse. | 1.00 |  |
| 8 | I believe that political participation to improve the health of the public is a natural duty of nurses. | 0.92 |  |
| 9 | I believe I am entitled to participate in activities to improve the healthcare system. | 0.92 |  |
| 10 | I believe that my participation in elections and voting behavior can have an important impact on society. | 0.75 | Revision |
| 11 | I believe that nurses' solidarity will increase the likelihood of political accomplishment. | 0.67 | Revision |
| 12 | I believe that politicians and public officials are working to improve the lives of people. | 0.75 | Revision |
| 13 | I am more interested in social issues and politics than other people. | 0.83 | Revision |
| 14 | I enjoy talking about political issues with others. | 0.83 |  |
| 15 | I have participated in activities (signatures, rallies, public hearings, etc.) of nursing professional organizations (Korean Nursing Association, Korean Nursing Association, various conferences, etc.). | 1.00 | Revision |
| 16 | I have met with external organizations (civil society organizations, political parties, public institutions, private institutions, etc.) to improve the healthcare system. | 0.83 | Revision |
| 17 | I have collaborated with external organizational groups to strengthen networking. | 1.00 | Revision |
| 18 | I have been a member of an external organizing group. | 0.92 | Revision |
| 19 | I have participated in the activities of a civil society organization or performed social service activities. | 0.83 |  |
| 20 | I respect the diversity of experiences and perceptions of others. | 0.83 |  |
| 21 | I am able to listen patiently to people whose political views are opposite to my own. | 1.00 | Revision |
| 22 | I can persuade coworkers to participate in political activities to improve the healthcare system. | 0.92 |  |
| 23 | I can meet with policymakers, such as politicians or government officials, to convince them to improve health care policy. | 1.00 | Revision |
| 24 | I can contribute to shaping public opinion in favor of health care reform. | 1.00 |  |
| 25 | I can explain my political views in a coherent way in speech or writing. | 1.00 |  |
| 26 | I have participated in all elections in the last two years. | 0.83 |  |
| 27 | I have posted comments on sites or social media related to political events or social issues. | 0.92 | Revision |
| 28 | I have voluntarily worked for a political party or candidate. | 0.83 |  |
| 29 | I have voluntarily contributed fund to a political party or candidate. | 0.83 |  |
| 30 | I am able to advocate for improvements in the nursing workplace. | 0.92 | Revision |
| 31 | I can advocate for an expanded role in nursing. | 1.00 | Revision |
| 32 | I have a vision for improving the healthcare system to benefit the health of the public. | 0.92 |  |
| 33 | I have a vision for the advancement of the nursing profession. | 0.92 |  |
| 34 | I am able to work collaboratively with others in the profession to improve the health of the public. | 0.92 |  |
| 35 | I believe it is important for nurses to be politically active to advocate for the public. | 1.00 |  |
| 36 | I am willing to work to improve the healthcare system in order to advocate for the public. | 1.00 |  |
| 37 | I am willing to solve problems in the nursing field to protect the rights of the public. | 1.00 |  |
| 38 | I have expressed my opinion on legislative and policy decisions to improve the health of the public. | 0.92 |  |

A preliminary survey was conducted to review the readability and clarity of the 39 preliminary items. The survey items were measured on a 4-point scale and average political competence score of the nurses who participated in the survey was 2.90±0.80 (Supplementary Table A7).

Table A7. Results of preliminary survey: participants’ political competence (N = 30)

| No. | Items | Mean±SD |
| --- | --- | --- |
| 1 | I know current political and social issues. | 3.37±0.76 |
| 2 | I know the recent main healthcare issues. | 3.17±0.83 |
| 3 | I know the healthcare policy-making process in my country. | 2.80±0.76 |
| 4 | I know the legislative processes in my country. | 2.60±0.77 |
| 5 | I gather information about major healthcare issues through various methods (TV, daily newspaper, internet, social media, nurses' newspaper. | 3.20±0.76 |
| 6 | I can systematically analyze information on key healthcare issues. | 2.21±0.56 |
| 7 | I can formulate reasonable alternatives to healthcare issues to improve public health. | 2.37±0.81 |
| 8 | I am proud to be a nurse. | 3.77±0.68 |
| 9 | I believe that political participation to improve public health is a natural duty of nurses. | 3.63±0.81 |
| 10 | I believe I have a responsibility and obligation to participate in activities to improve the healthcare system. | 3.57±0.90 |
| 11 | I believe that my participation in elections and voting behavior can have an important impact on society. | 4.10±0.71 |
| 12 | I believe that nurses' solidarity will increase the likelihood of political change. | 3.73±0.69 |
| 13 | I believe that politicians or government officials are working to improve the lives of the public. | 2.77±0.97 |
| 14 | I am interested in social issues and politics. | 3.40±0.86 |
| 15 | I enjoy talking with others about political issues. | 2.77±0.97 |
| 16 | I have participated in activities (seminars, public hearings, signatures, rallies or events, etc.) of nursing professional organizations (such as the Korean Nursing Association, the Korean Nursing Fellowship, various conferences, etc. | 2.80±1.06 |
| 17 | I have met with external organizations (civil society organizations, political parties, public institutions, private institutions, etc.) to improve the healthcare system. | 2.27±0.78 |
| 18 | I have collaborated with an external organization to strengthen networking. | 2.07±0.69 |
| 19 | I have been a member of an external organizing group. | 1.97±0.76 |
| 20 | I have participated in the activities of a civil society organization or performed community service. | 2.73±0.98 |
| 21 | I respect the diversity of experiences and perceptions of others. | 3.67±0.61 |
| 22 | I am able to listen patiently to people whose political views are opposite to my own. | 3.27±0.83 |
| 23 | I am willing to actively explain to my coworkers and acquaintances how to get involved in political activities to improve the healthcare system. | 2.87±0.82 |
| 24 | I can meet with policymakers, such as politicians or government officials, and explain health policy improvements in a logical way. | 2.67±0.81 |
| 25 | I can contribute to shaping public opinion in favor of improving the healthcare system. | 2.63±0.72 |
| 26 | I can explain my political views in a coherent manner in speech or writing. | 2.47±0.73 |
| 27 | I have participated in all elections in the last two years. | 4.13±0.82 |
| 28 | I have experience posting comments on sites or social media related to political or social issues. | 1.90±0.84 |
| 29 | I have voluntarily worked for a political party or candidate. | 1.60±0.62 |
| 30 | I have voluntarily contributed fund to a political party or candidate. | 1.63±0.72 |
| 31 | I can advocate for improved working conditions for nurses. | 3.47±0.86 |
| 32 | I can advocate for an expanded role for nurses. | 3.43±0.86 |
| 33 | I have a vision for improving the healthcare system to benefit the health of the public. | 2.87±0.78 |
| 34 | I have a vision for the advancement of the nursing profession. | 3.10±0.80 |
| 35 | I can collaborate with others in other professions in political activities to improve the health of the public. | 2.53±1.01 |
| 36 | I believe it is important for nurses to be politically active to advocate on behalf of the public. | 3.77±0.73 |
| 37 | I am willing to work to improve the health care system in order to advocate for the public. | 3.07±0.87 |
| 38 | I am committed to solving problems in nursing practice to secure the right to health for the people. | 3.10±0.80 |
| 39 | I have provided input into legislative and policy decisions to improve the health of the public. | 1.90±0.96 |

The average time required to fill out the questionnaire was 10.63±3.84 minutes, the appropriateness of the font size was 3.33 ± 0.55, the appropriateness of the item length was 3.17 ± 0.46, and the appropriateness of the response score size (5-point scale) was 3.23 ± 0.50 (Supplementary Table A8).

Table A8. Results of preliminary survey: the readability and clarity of the 39 preliminary items (N = 30)

| No | Classification | Range | Mean±SD |
| --- | --- | --- | --- |
| 1 | Time taken to complete the questionnaire | 3-20 | 10.63±3.84 |
| 2 | Appropriateness of the item font size | 1-4 | 3.33±0.55 |
| 3 | Appropriateness of each item length | 1-4 | 3.17±0.46 |
| 4 | Appropriateness of response score size (5-point scale) | 1-4 | 3.23±0.50 |
| 5 | Items that you don't understand and reasons | No response | |
| 6 | Items that must be deleted and their reasons | No response | |
| 7 | Other comments requiring revision of the items | No response | |

**Primary tool test (1^st^ survey): 39 items -> 38 items -> 35 items**

**Item-total Correlations of Preliminary Items**

The average score for each 39 preliminary items ranged from 1.88 to 4.11. The absolute value of skewness ranged from 0.029 to 1.160, and the kurtosis value ranged from 0.041 to 1.263, confirming that all items had a normal distribution. As a result of analyzing the corrected item-total coefficient, one item was deleted because it was less than r=.30. The average of Alpha if item deleted to identify items that threaten reliability was .937, and all items were found to be within the appropriate range of .935 to .940 (Supplementary Table A9).

Table A9. The results of Item-total Correlations of Preliminary Items (N = 224)

| No. | Items | Mean±SD | Skewness | Kurtosis | Corrected item-total correlation | Alpha if item deleted |
| --- | --- | --- | --- | --- | --- | --- |
| 1 | I know current political and social issues. | 3.35±0.77 | -0.278 | 0.275 | .522 | .937 |
| 2 | I know the recent main healthcare issues. | 3.17±0.78 | -0.032 | -0.518 | .578 | .937 |
| 3 | I know the healthcare policy-making process in my country. | 2.71±0.84 | 0.254 | -0.305 | .568 | .937 |
| 4 | I know the legislative processes in my country. | 2.65±0.82 | 0.344 | -0.162 | .512 | .937 |
| 5 | I gather information about major healthcare issues through various methods (TV, daily newspaper, internet, social media, nurses' newspaper. | 3.09±0.90 | -0.035 | -0.771 | .438 | .938 |
| 6 | I can systematically analyze information on key healthcare issues. | 2.40±0.73 | 0.150 | -0.198 | .582 | .937 |
| 7 | I can formulate reasonable alternatives to healthcare issues to improve public health. | 2.45±0.76 | 0.121 | -0.297 | .532 | .937 |
| 8 | I am proud to be a nurse. | 3.67±0.74 | -0.279 | -0.094 | .439 | .938 |
| 9 | I believe that political participation to improve public health is a natural duty of nurses. | 3.72±0.84 | -0.117 | -0.617 | .506 | .937 |
| 10 | I believe I have a responsibility and obligation to participate in activities to improve the healthcare system. | 3.74±0.80 | -0.458 | 0.476 | .533 | .937 |
| 11 | I believe that my participation in elections and voting behavior can have an important impact on society. | 4.11±0.83 | -0.789 | 0.474 | .407 | .938 |
| 12 | I believe that nurses' solidarity will increase the likelihood of political change. | 3.64±0.88 | -0.242 | -0.409 | .411 | .938 |
| 13 | I believe that politicians or government officials are working to improve the lives of the public. | 2.92±0.95 | -0.008 | -0.205 | .344 | .939 |
| 14 | I am interested in social issues and politics. | 3.29±0.82 | 0.119 | -0.295 | .649 | .936 |
| 15 | I enjoy talking with others about political issues. | 2.83±0.90 | 0.254 | -0.141 | .575 | .937 |
| 16 | I have participated in activities (seminars, public hearings, signatures, rallies or events, etc.) of nursing professional organizations (such as the Korean Nursing Association, the Korean Nursing Fellowship, various conferences, etc. | 2.67±1.11 | 0.237 | -0.867 | .474 | .938 |
| 17 | I have met with external organizations (civil society organizations, political parties, public institutions, private institutions, etc.) to improve the healthcare system. | 1.97±0.90 | 0.742 | 0.060 | .474 | .937 |
| 18 | I have collaborated with an external organization to strengthen networking. | 2.01±0.91 | 0.777 | 0.134 | .531 | .937 |
| 19 | I have been a member of an external organizing group. | 1.88±0.91 | 1.099 | 0.928 | .472 | .937 |
| 20 | I have participated in the activities of a civil society organization or performed community service. | 2.33±1.70 | 0.472 | -0.694 | .482 | .938 |
| 21 | I respect the diversity of experiences and perceptions of others. | 4.00±0.66 | -0.186 | -0.131 | .416 | .938 |
| 22 | I am able to listen patiently to people whose political views are opposite to my own. | 3.64±0.72 | -0.277 | 0.335 | .381 | .938 |
| 23 | I am willing to actively explain to my coworkers and acquaintances how to get involved in political activities to improve the healthcare system. | 2.94±0.88 | -0.240 | -0.091 | .654 | .936 |
| 24 | I can meet with policymakers, such as politicians or government officials, and explain health policy improvements in a logical way. | 2.50±0.92 | 0.408 | -0.041 | .654 | .936 |
| 25 | I can contribute to shaping public opinion in favor of improving the healthcare system. | 2.82±0.94 | -0.055 | -0.373 | .691 | .935 |
| 26 | I can explain my political views in a coherent manner in speech or writing. | 2.57±0.79 | 0.094 | -0.106 | .620 | .936 |
| 27 | I have participated in all elections in the last two years. | 4.11±0.98 | -0.918 | 0.186 | .168 | .940 |
| 28 | I have experience posting comments on sites or social media related to political or social issues. | 2.11±1.01 | 0.849 | 0.105 | .468 | .938 |
| 29 | I have voluntarily worked for a political party or candidate. | 1.80±0.88 | 1.160 | 1.263 | .414 | .938 |
| 30 | I have voluntarily contributed fund to a political party or candidate. | 1.97±1.06 | 1.072 | 0.446 | .381 | .939 |
| 31 | I can advocate for improved working conditions for nurses. | 3.34±0.93 | -0.285 | -0.246 | .479 | .937 |
| 32 | I can advocate for an expanded role for nurses. | 3.32±0.93 | -0.029 | -0.399 | .578 | .937 |
| 33 | I have a vision for improving the healthcare system to benefit the health of the public. | 2.79±0.85 | 0.151 | -0.066 | .718 | .935 |
| 34 | I have a vision for the advancement of the nursing profession. | 2.80±0.87 | 0.362 | -0.184 | .679 | .936 |
| 35 | I can collaborate with others in other professions in political activities to improve the health of the public. | 2.66±0.90 | 0.313 | -0.059 | .703 | .935 |
| 36 | I believe it is important for nurses to be politically active to advocate on behalf of the public. | 3.71±0.87 | -0.368 | -0.062 | .484 | .937 |
| 37 | I am willing to work to improve the health care system in order to advocate for the public. | 3.20±0.89 | -0.124 | -0.165 | .654 | .936 |
| 38 | I am committed to solving problems in nursing practice to secure the right to health for the people. | 3.11±0.83 | -0.118 | 0.043 | .614 | .936 |
| 39 | I have provided input into legislative and policy decisions to improve the health of the public. | 2.16±1.00 | 0.861 | 0.510 | .475 | .937 |

**Construct Validity: 1^st^ Exploratory Factor Analysis (EFA)**

EFA was conducted after the Item-total Correlations test to identify the construct validity for 38 items. KMO and Bartlett's test of sphericity results for 38 items were KMO=.900, and Battlett's sphericity test value was also significant ($x^{2}$=5038.197 (*p*<.000), suitable for EFA. As a result of EFA, eight factors according to Kaiser's criterion (1974) with an eigenvalue of 1 or more (Supplementary Table A10).

Table A10. Factor loading from exploratory factor analysis (38 items): 1^ST^ EFA (N = 224)

| Items | Communalities | FactorⅠ | FactorⅡ | Factor Ⅲ | Factor Ⅳ | Factor Ⅴ | Factor Ⅵ | Factor Ⅶ | Factor Ⅷ |
| --- | --- | --- | --- | --- | --- | --- | --- | --- | --- |
| 9 | .788 | .835 | .114 | .176 | -.059 | .191 | .083 | .030 | -.006 |
| 10 | .732 | .789 | .116 | .147 | .047 | .262 | .063 | -.001 | -.015 |
| 8 | .522 | .655 | .178 | .074 | .074 | .087 | .030 | -.017 | .204 |
| 12 | .545 | .627 | .087 | .168 | .042 | -.105 | .293 | .035 | .126 |
| 11 | .576 | .534 | .071 | .323 | -.043 | -.185 | .177 | .193 | .277 |
| 36 | .583 | .507 | .030 | .380 | .023 | .067 | .345 | -.117 | .209 |
| 37 | .669 | .460 | .044 | .446 | .107 | .371 | .312 | -.003 | .104 |
| 7 | .702 | .144 | .753 | .087 | .124 | .246 | -.104 | -137 | -.020 |
| 3 | .688 | .045 | .734 | .058 | .206 | .157 | .215 | .050 | .166 |
| 6 | .685 | .030 | .727 | .162 | .188 | .279 | .019 | .124 | -.014 |
| 4 | .641 | .018 | .697 | .157 | .078 | .072 | .296 | -.026 | .179 |
| 2 | .649 | .227 | .636 | .108 | .246 | -.003 | .345 | .032 | .004 |
| 5 | .407 | .274 | .521 | .129 | .074 | -.040 | .126 | .141 | -.037 |
| 26 | .557 | .041 | .428 | .370 | .118 | .427 | .155 | .084 | .086 |
| 32 | .775 | .236 | .113 | .813 | .006 | .103 | .106 | .099 | .121 |
| 31 | .741 | .078 | .077 | .792 | .007 | -.020 | .150 | .127 | .253 |
| 33 | .757 | .255 | .271 | .674 | .235 | .328 | .015 | .037 | .007 |
| 34 | .731 | .300 | .231 | .669 | .238 | .245 | .010 | .108 | -.111 |
| 35 | .670 | .279 | .288 | .570 | .202 | .337 | .040 | .156 | -.061 |
| 19 | .751 | .016 | .087 | .045 | .812 | .193 | .149 | .131 | -.079 |
| 17 | .743 | -.050 | .210 | -.012 | .780 | .201 | .046 | .203 | .067 |
| 18 | .707 | .033 | .153 | .083 | .747 | .236 | .007 | .229 | .096 |
| 20 | .632 | -.084 | .132 | .160 | .708 | .217 | .078 | .107 | -.126 |
| 16 | .611 | .243 | .229 | .176 | .682 | -.023 | .010 | -.054 | -.003 |
| 24 | .765 | .046 | .369 | .107 | .215 | .701 | .126 | .217 | .122 |
| 39 | .618 | -.023 | .099 | .121 | .202 | .684 | .023 | .290 | .000 |
| 25 | .679 | .131 | .245 | .275 | .238 | .624 | .157 | .188 | .139 |
| 23 | .624 | .230 | .256 | .189 | .229 | .572 | .128 | .214 | .264 |
| 38 | .563 | .275 | .116 | .418 | .237 | .463 | .046 | -.055 | .152 |
| 13 | .372 | .268 | -.026 | .011 | .259 | .407 | .176 | -.188 | -.009 |
| 14 | .796 | .296 | .240 | .212 | .068 | .287 | .716 | .070 | .025 |
| 15 | .714 | .176 | .272 | .037 | .121 | .331 | .686 | .108 | .046 |
| 1 | .643 | .257 | .377 | .139 | .116 | -.063 | .613 | .122 | .081 |
| 29 | .783 | -.039 | .160 | .084 | .204 | .246 | .055 | .796 | -.099 |
| 30 | .656 | .109 | .134 | .067 | .179 | .065 | .016 | .760 | .086 |
| 28 | .605 | -.064 | .047 | .315 | .167 | .309 | .247 | .556 | -.077 |
| 22 | .755 | .119 | .067 | .088 | .099 | .232 | .145 | -.037 | .802 |
| 21 | .706 | .414 | .125 | .209 | .042 | .045 | -.062 | -.008 | .684 |
| Eigenvalue | | 4.018 | 4.012 | 3.882 | 3.616 | 3.592 | 2.254 | 2.112 | 1.659 |
| % of variance | | 10.574 | 10.557 | 10.216 | 9.515 | 9.452 | 5.932 | 5.557 | 4.365 |
| Cumulative (%) | | 10.574 | 21.131 | 31.347 | 40.861 | 50.313 | 56.245 | 61.802 | 66.168 |

The number of factors right before the slope of the Scree Plot started to become gentle appeared somewhat ambiguous (n=4~8) (Supplementary Figure A2).


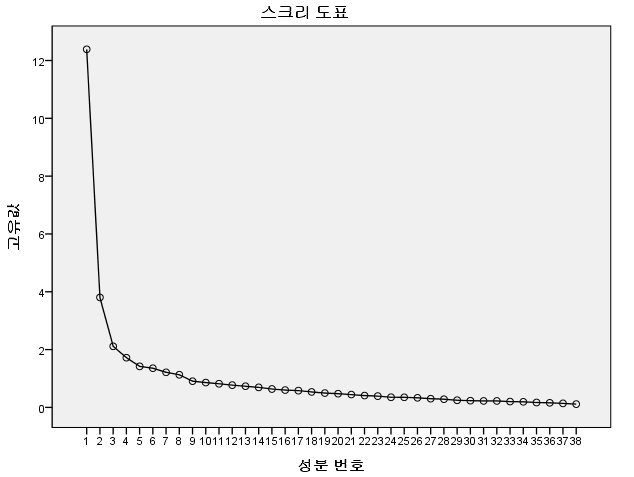


Figure A2. The result of 1^st^ EFA: scree plot (38 items)

**Construct Validity: Parallel Analysis**

Parallel Analysis was performed to supplement this, and the number of factors for which the eigenvalues analyzed from the actual data were greater than the eigenvalues of the randomly generated data was four factors. Therefore, extracting the four factors based on the results of Kaiser's criterion, scree Plot, and Parallel Analysis was identified as most appropriate (Supplementary Table A11, Supplementary Figure A3).

Table A11. The result of the Parallel Analysis (38 items)

| Principle Components & Random Normal Data Generation | | | |
| --- | --- | --- | --- |
| Specifications for this Run: | | | |
| N cases |  |  |  |
| N var |  |  |  |
| N datsets |  |  |  |
| Percent |  |  |  |
| Raw Data Eigenvalues, & Mean & Percentile Random Data Eigenvalues | | | |
| Root | Raw Data Eigenvalues | Means | Percentile |
| 1.00000000 | 12.388 | 1.88194836 | 1.99876200 |
| 2.00000000 | 3.805 | 1.77127423 | 1.84957082 |
| 3.00000000 | 2.114 | 1.68904902 | 1.75729756 |
| 4.00000000 | 1.720 | 1.61782459 | 1.67823397 |
| 5.00000000 | 1.420 | 1.55775332 | 1.61006981 |
| 6.00000000 | 1.356 | 1.50081648 | 1.55303543 |
| 7.00000000 | 1.213 | 1.44723470 | 1.49339086 |
| 8.00000000 | 1.129 | 1.39632608 | 1.44072422 |

**Construct Validity: 2^nd^ Exploratory Factor Analysis’ Scree plot**


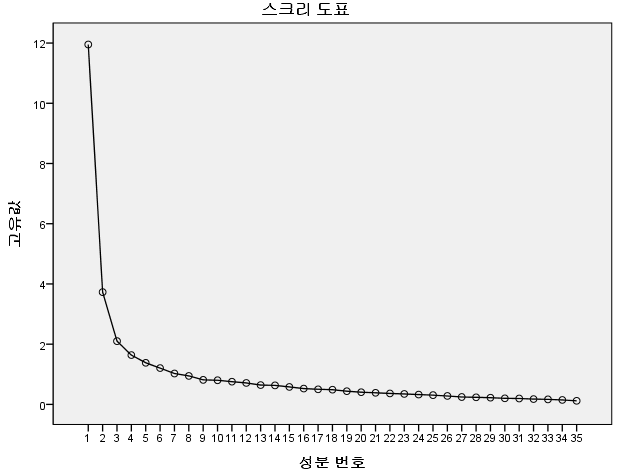


Figure A3. The result of 2^ND^ EFA: scree plot (35 items)

**Convergent and Discriminative Validity**

Through Multi-trait/multi-item matrix analysis, Convergent Validity showed that the correlation coefficient of each factor was *r*=.459 to *r*=.752, and all 35 items were *r*=.40 or higher, meeting the test criteria. Discriminant Validity was greater than the standard error value of 2 times for all 35 items, so it met the test criteria (Supplementary Table A12).

Table A12. The results of the Multi-trait/multi-item matrix (35 items) (N=224)

| The correlation matrix corrected for overlap | | | | | |
| --- | --- | --- | --- | --- | --- |
| Items | FactorⅡ | Factor Ⅲ | Factor Ⅳ | Factor Ⅴ | r-2SE |
| 8 | .569 | .321 | .354 | .166 | 0.463 |
| 9 | .736 | .408 | .385 | .096 | 0.676 |
| 10 | .690 | .434 | .390 | .192 | 0.608 |
| 11 | .569 | .314 | .303 | .071 | 0.469 |
| 12 | .595 | .276 | .349 | .111 | 0.497 |
| 21 | .505 | .323 | .279 | .149 | 0.405 |
| 36 | .641 | .384 | .373 | .126 | 0.551 |
| 37 | .630 | .628 | .460 | .284 | 0.542 |
|  |  |  |  |  |  |
| 23 | .448 | .640 | .461 | .454 | 0.542 |
| 24 | .287 | .681 | .565 | .480 | 0.603 |
| 25 | .406 | .720 | .531 | .487 | 0.652 |
| 26 | .359 | .626 | .556 | .374 | 0.53 |
| 28 | .171 | .540 | .352 | .366 | 0.442 |
| 29 | .061 | .459 | .325 | .381 | 0.329 |
| 31 | .426 | .507 | .323 | .163 | 0.405 |
| 32 | .553 | .612 | .379 | .196 | 0.522 |
| 33 | .526 | .744 | .498 | .417 | 0.674 |
| 34 | .524 | .693 | .459 | .402 | 0.625 |
| 35 | .491 | .725 | .520 | .406 | 0.655 |
| 38 | .485 | .601 | .408 | .388 | 0.509 |
| 39 | .153 | .562 | .321 | .406 | 0.466 |
|  |  |  |  |  |  |
| 1 | .445 | .387 | .596 | .253 | 0.506 |
| 2 | .380 | .420 | .690 | .383 | 0.618 |
| 3 | .298 | .454 | .680 | .395 | 0.608 |
| 4 | .301 | .404 | .650 | .285 | 0.578 |
| 5 | .322 | .342 | .481 | .227 | 0.415 |
| 6 | .234 | .536 | .640 | .421 | 0.576 |
| 7 | .264 | .476 | .589 | .357 | 0.525 |
| 14 | .555 | .547 | .627 | .282 | 0.557 |
| 15 | .408 | .480 | .591 | .328 | 0.511 |
|  |  |  |  |  |  |
| 16 | .296 | .377 | .383 | .540 | 0.464 |
| 17 | .087 | .436 | .381 | .752 | 0.662 |
| 18 | .181 | .485 | .369 | .715 | 0.625 |
| 19 | .117 | .426 | .353 | .734 | 0.632 |
| 20 | .127 | .479 | .345 | .659 | 0.579 |

**Internal Consistency Reliability**

The result of calculating the Cronbach's α of all items to test the internal consistency was .940, and the Cronbach's α for each of the four factors ranged from .856 to .907, meeting the test standard that the newly developed scale should meet .70 or higher (Supplementary Table A13).

Table A13. Internal Consistency Reliability of Political Competence Scale for nurses (N=224)

| Factors |  | Number of items | Cronbach’s α |
| --- | --- | --- | --- |
|  |  | 35 | .940 |
| Ⅰ(Political Efficacy) |  | 8 | .866 |
| Ⅱ(Political Activity) |  | 13 | .907 |
| Ⅲ(Political Knowledge) |  | 9 | .874 |
| Ⅳ(Political Interaction) |  | 5 | .856 |

**Correlation among Factors**

The results of the correlation analysis among the factors of the tool showed a statistically significant and relatively high correlation: ‘political activity’ and ‘political knowledge’ *r*=.633 (*p*<.001), ‘political activity’ and ‘political interaction’ *r*=.549 (*p*<.001), ‘political efficacy’ and ‘political activity’ *r*=.541 (*p*<.001), and ‘political efficacy’ and political knowledge’ *r*=.506 (*p*<.001). A statistically significant relatively low correlation was shown with ‘political efficacy’ and ‘political interaction’ *r*=.208 (*p*=.002) (Supplementary Table A14).

Table A14. Correlations among factors of Political Competence Scale for nurses (N=224)

| Factors | FactorⅡ | Factor Ⅲ | Factor Ⅳ | Factor Ⅴ |
| --- | --- | --- | --- | --- |
|  | r (*p*) | r (*p*) | r (*p*) | r (*p*) |
| Ⅰ(Political Efficacy) | 1 |  |  |  |
| Ⅱ(Political Activity) | .541 (<.001) | 1 |  |  |
| Ⅲ(Political Knowledge) | .506 (<.001) | .633 (<.001) | 1 |  |
| Ⅳ(Political Interaction) | .208 (.002) | .549 (<.001) | .457 (<.001) | 1 |

**Secondary tool test (2^nd^ survey): Confirm 35 items and four factors**

As the reliability and validity of the tool consisting of four factors and 35 items were established, confirmatory factor analysis, criterion validity, and internal consistency reliability were analyzed to test the model fit of the political competence Scale-Nurses (PCS-N).

**Verification of Known-groups Validity**

As a result of the Known-group Validity analysis, at the significance level of p<.05, the group that currently or in the past joined a political party is better than the non-member group (F=3.36, p=0.036), the group that is currently or in the past member of the Korea Nursing Association is better than the non-member group (F=6.721, p<.001), the group that has currently or in the past joined a volunteer group is better than the non-member group (F=11.078, p<.001), citizens The group that was presently or in the past joined a social group had a statistically significantly higher level of political competence than the group that did not (F=11.094, p<.001) (Supplementary Table A15).

Table A15. Verification of Known-groups Validity (N=225)

| Variables | | Categories | N (%) | Political Competence | | |
| --- | --- | --- | --- | --- | --- | --- |
|  |  |  |  | Mean±SD | F(*p)* | Scheffe |
| Joining parties |  | At present | 9(4.00) | 3.06±0.72 | 3.360 | 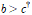 |
|  |  | In the past but not currently joined | 18(8.00) | 3.21±0.38 | (.036) |  |
|  |  | Never joined | 198(88.00) | 2.89±0.52 |  |  |
| Joining nursing organizations |  | At present | 24(10.67) | 3.06±0.55 | 6.721 | 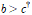 |
|  |  | In the past but not currently joined | 13(5.78) | 3.37±0.42 | (.001) |  |
|  |  | Never joined | 188(83.56) | 2.87±0.51 |  |  |
| Joining service groups |  | At present | 26(11.56) | 3.17±0.54 | 11.078 | 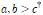 |
|  |  | In the past but not currently joined | 23(10.22) | 3.27±0.63 | (<.001) |  |
|  |  | Never joined | 176(78.22) | 2.84±0.47 |  |  |
| Joining civic groups |  | At present | 4(1.78) | 3.52±0.73 | 11.094 | 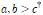 |
|  |  | In the past but not currently joined | 6(2.67) | 3.73±0.83 | (<.001) |  |
|  |  | Never joined | 215(95.56) | 2.89±0.49 |  |  |

†post-hoc (Scheffe) test
