# Supplementary material for: The Political Competence Scale for Nurses (PCS-N): Instrument Development and Psychometric Evaluation
Source: J Nurs Manag. 2025 May 19;2025:4683994. doi: 10.1155/jonm/4683994 (PMC12105907; doi:10.1155/jonm/4683994)
Supplement: Supporting Information 2 — Supporting Information B includes the Political Competence Scale for Nurses (PCS-N) items verified for reliability, validity, and model fit. [file 4683994.f2.pdf]

Supplementary material B (English version)

Political Competence Scale for Nurses (PCS-N)

This questionnaire includes 35 items to measure nurses' political competence. Its measurement scale consists of four factors: Political Knowledge, Political Efficacy, Political Interaction, and Political Activity. Your responses will be confidential, so please be as open and honest as possible. I appreciate your cooperation.

**1**	**2**	**3**	**4**	**5**
Strongly disagree	Disagree	Neutral	Agree	Strongly agree

Please read the following questions and circle the number most closely fits your opinion.

Political Knowledge						
PC1	I know current issues related to politics or society.	1	2	3	4	5
PC2	I know current major healthcare issues.	1	2	3	4	5
PC3	I know the healthcare policy decision-making process in my country.	1	2	3	4	5
PC4	I know the legislative process in my country.	1	2	3	4	5
PC5	I collect information on healthcare-related issues through various media (TV, daily newspaper, Internet, SNS, nursing-related newspapers or newsletters, etc.).	1	2	3	4	5
PC6	I can systematically analyse information on healthcare issues.	1	2	3	4	5
PC7	I can establish reasonable alternatives to healthcare issues to promote public health.	1	2	3	4	5
PC8	I am interested in social issues or politics.	1	2	3	4	5
PC9	I enjoy talking to people around me about political issues.	1	2	3	4	5
Political Efficacy						
PC10	I am proud to be a nurse.	1	2	3	4	5
PC11	I think that political participation to improve public health is a natural duty of nurses.	1	2	3	4	5
PC12	I believe that I have a responsibility and duty to participate in activities to improve the healthcare system.	1	2	3	4	5
PC13	I believe that my participation in elections or voting can significantly impact society.	1	2	3	4	5
PC14	I believe that nurses' solidarity will increase the possibility of political success.	1	2	3	4	5
PC15	I respect the diversity of experiences and perceptions of others.	1	2	3	4	5
PC16	I believe it is important for nurses to be interested in political activities to represent the people.	1	2	3	4	5
PC17	I have the will to promote healthcare system improvement to advocate for the people.	1	2	3	4	5

Political Interaction						
PC18	I have experience participating in political activities (seminars, public hearings, signatures, rally, events, etc.)	1	2	3	4	5
PC19	I have experience meeting with members of external organizations (civil society groups, political parties, public institutions, private institutions, etc.) to improve the healthcare system.	1	2	3	4	5
PC20	I have experience working with external organizations to strengthen political networking.	1	2	3	4	5
PC21	I have been a member of external organizations (civil society groups, political parties, public institutions, private institutions, etc.)	1	2	3	4	5
PC22	I have experience participating in activities of civil society organizations or doing community service activities.	1	2	3	4	5
Political Activity						
PC23	I have the will to actively explain to the people around me to participate in political activities to improve the healthcare system.	1	2	3	4	5
PC24	I can logically explain healthcare policy improvement by meeting policymakers such as politicians or civil servants.	1	2	3	4	5
PC25	I can contribute to forming public opinion for improving the health care system.	1	2	3	4	5
PC26	I can logically explain my political views verbally and in writing.	1	2	3	4	5
PC27	I have experience posting opinions on internet or SNS related to political or social issues.	1	2	3	4	5
PC28	I have experience voluntarily working for a particular political party or candidate.	1	2	3	4	5
PC29	I can demand improvements in the working environment of nurses.	1	2	3	4	5
PC30	I can argue for the expansion of the role of nurses.	1	2	3	4	5
PC31	I have a vision for improving the healthcare system that is promotive to public health.	1	2	3	4	5
PC32	I have a vision for the advancement of the nursing profession.	1	2	3	4	5
PC33	I can engage in political activities in cooperation with people in other occupations to improve public health.	1	2	3	4	5
PC34	I strive to solve problems that occur in nursing field to secure public right to health.	1	2	3	4	5
PC35	I have opinions on legislation and policy decisions to improve public health.	1	2	3	4	5
